# Synthesis and Molecular Modelling Studies of New 1,3-Diaryl-5-Oxo-Proline Derivatives as Endothelin Receptor Ligands

**DOI:** 10.3390/molecules25081851

**Published:** 2020-04-17

**Authors:** Sebastiano Intagliata, Mohamed A. Helal, Luisa Materia, Valeria Pittalà, Loredana Salerno, Agostino Marrazzo, Alfredo Cagnotto, Mario Salmona, Maria N. Modica, Giuseppe Romeo

**Affiliations:** 1 Department of Drug Sciences, University of Catania, Viale A. Doria 6, 95125 Catania, Italy; s.intagliata@unict.it (S.I.); vpittala@unict.it (V.P.); lsalerno@unict.it (L.S.); marrazzo@unict.it (A.M.); gromeo@unict.it (G.R.); 2 University of Science and Technology, Biomedical Sciences Program, Zewail City of Science and Technology, October Gardens, 6th of October, Giza 12578, Egypt; mhelal@zewailcity.edu.eg; 3 Medicinal Chemistry Department, Faculty of Pharmacy, Suez Canal University, Ismailia 41522, Egypt; 4 Istituto di Ricerche Farmacologiche “Mario Negri”, IRCCS. Via Mario Negri, 2, 20156 Milano, Italy; alfredo.cagnotto@marionegri.it (A.C.); mario.salmona@marionegri.it (M.S.)

**Keywords:** endothelin receptors, ET_A_ ligands, 1,3-diaryl-5-oxo-proline derivatives, homology modeling, molecular docking

## Abstract

The synthesis of seventeen new 1,3-diaryl-5-oxo-proline derivatives as endothelin receptor (ETR) ligands is described. The structural configuration of the new molecules was determined by analyzing selected signals in proton NMR spectra. In vitro binding assays of the human ET_A_ and ET_B_ receptors allowed us to identify compound **31h** as a selective ET_A_R ligand. The molecular docking of the selected compounds and the ET_A_ antagonist atrasentan in the ET_A_R homology model provided insight into the structural elements required for the affinity and the selectivity of the ET_A_R subtype.

## 1. Introduction

**Figure 1:**
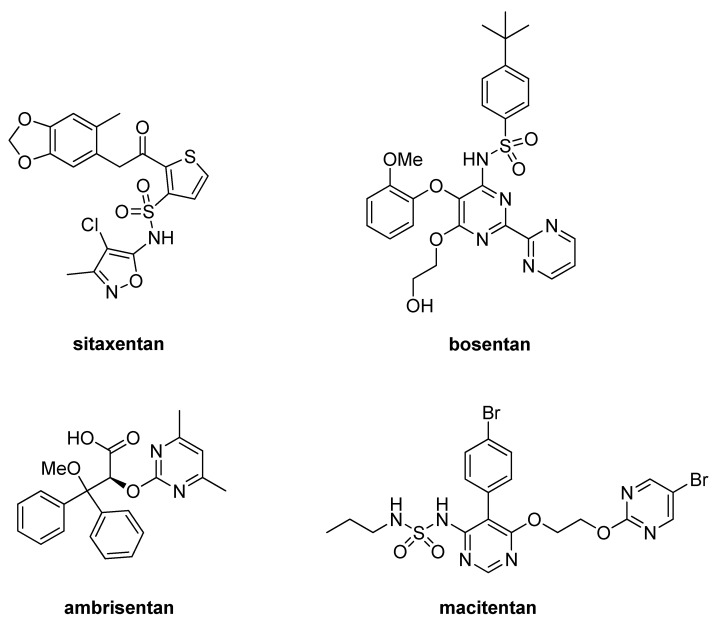
Marketed Endothelin (ET) antagonists.

Endothelins (ETs) are a group of peptides involved in vascular homeostasis [1]. Three different isoforms have been identified and are named endothelin-1, -2, and -3 (ET-1, ET-2, and ET-3) [1]. ET-1 is a potent vasoconstrictor agent that was first isolated from porcine aortic endothelial cells in 1985 and structurally characterized in 1988 by Yanagisawa et al. [2]. Successively, two other peptides, ET-2 and ET-3, were identified in humans [3]. These three endothelin isoforms interact with different endothelin receptors (ETRs) and both subtypes, ET_A_R and ET_B_R, are G protein-coupled receptors (GPCRs). The endogenous ligands bind the ETRs with different potencies. ET-1 and ET-2 possess higher affinities towards ET_A_R compared to ET-3 [4], while all three ET isoforms are equally effective for ET_B_R [5]. Both the ETRs are distributed in several tissues, including vascular smooth muscle and endothelial cells. High plasma concentrations in ETs have been observed in certain pathological conditions where excessive vasoconstriction or smooth muscle proliferation occurs (e.g., systemic hypertension, atherosclerosis, and pulmonary hypertension). These discoveries have aroused great interest among academics and in the pharmaceutical industry, leading to the development of a large number of ETR ligands, especially ET_A_ antagonists, which are useful as antihypertensive drugs. Some ETR ligands, including sitaxentan (ET_A_ selective antagonist), bosentan (mixed antagonist), ambrisentan (ET_A_ selective antagonist), and macitentan (mixed antagonist), have been marketed and are currently used as medications to treat pulmonary arterial hypertension (PAH) (Figure 1). Among them, sitaxentan was later removed from the market in some countries because of its high liver toxicity.

**Figure 2:**
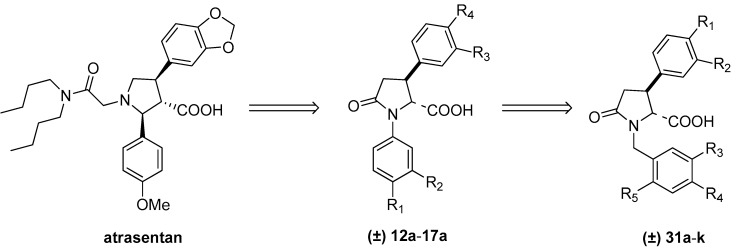
Chemical structures of atrasentan and the newly synthesized ligands.

Different structure–activity relationship (SAR) studies among several classes of ligands have allowed us to elucidate the main structural elements required for binding of the ETRs. For example, two aromatic portions and a carboxylic acid function, adequately oriented in the space between the aromatic portions, constitute a common scaffold for many active compounds [6,7,8]. Moreover, the presence of aromatic portions, which are directly (or through methylene, ether, or thioether functions) linked to the central nucleus and substituted with electron-donating groups, generally promote receptor binding. The selective ET_A_ antagonist atrasentan (Figure 2) is an excellent example of a compound possessing the structural features mentioned above. Preliminary in vivo studies demonstrated that selective ET_A_ antagonists counteract the fibrotic, inflammatory, and proliferative action of ET-1 at the kidney level [9]. These data support the clinical evaluation of ET_A_ antagonists, including atrasentan, as renoprotective agents for diabetic kidney disease [9,10]. Unfortunately, the phase III clinical study of diabetic nephropathy with atrasentan (named SONAR) was closed in advance due to the considerably fewer endpoints obtained than expected by that time. Moreover, atrasentan has shown some adverse effects, such as fluid retention and an increased risk of hospitalization for heart failure [11].

Advancement in molecular biology has provided important techniques that play a pivotal role in the modern drug development process [12], allowing the discovery of several new drug targets, especially in oncology [13,14,15,16,17,18]. On the other hand, drug repurposing is becoming a more and more attractive approach among pharmaceutical companies due to the clear advantages in cost and time reduction compared to de novo drug development [19,20]. Examples of the re-tasking of known medications and clinical candidates for new medical indications include disulfiram, valproic acid, and the already mentioned atrasentan. For selective ET_A_R antagonists, more recent studies have reported a correlation between the overexpression of endothelin receptors and some solid cancers, such as ovarian, breast, and prostate cancer [21,22,23]. Moreover, ET_A_ antagonists have shown clear effects on tumor proliferation, apoptosis, and angiogenesis [24]. Therefore, ET_A_ antagonists have experienced renewed interest in their potential anticancer applications. For example, atrasentan has been clinically evaluated both alone and in combination with anticancer agents for the treatment of various types of cancer, including advanced prostate cancer and non-small cell lung cancer [25,26,27]. Despite considerable efforts to translate atrasentan into the market, and after several years of different clinical evaluations, no approval from regulatory agencies has yet been obtained. Thus, the development of new selective ET_A_R ligands, which may serve as successful chemical probes for the discovery of new cancer drugs, is needed.

Within the framework of our studies on ETR ligands [28,29,30], the present paper reports the synthesis of new 1,3-diaryl-5-oxo-proline derivatives, compounds (±)-*trans* **12a**–**17a**, obtained as a racemic mixture (Figure 2). The newly synthesized compounds possess a 1,3-biaryl substituted 5-oxo-proline core in which the carboxylic acid function and the aromatic substituent at the 3-position are in the *trans* configuration. Furthermore, compounds (±)-*trans* **31a**–**k,** bearing a tri-substituted benzyl group at the nitrogen atom of the pyrrolidine ring, were also prepared to obtain more information on the receptor–ligand interactions (Figure 2). Finally, we developed a homology model of ET_A_R and performed molecular docking to investigate the binding mode of atrasentan, **15a**, and the selective analog, **31h**.

## 2. Results and Discussion

### 2.1. Chemistry

**Scheme 1:**
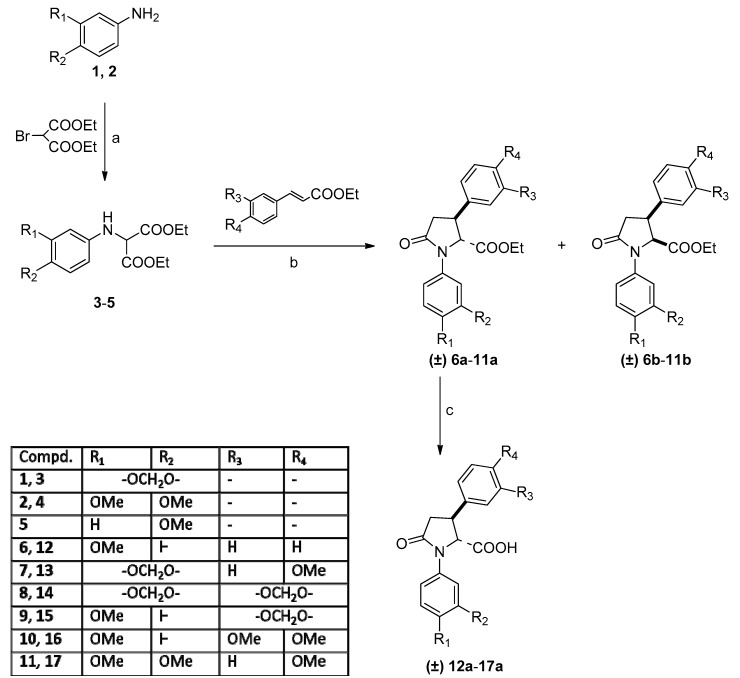
Reagents and conditions: (**a**), room temperature 3 days; (**b**) EtONa, dry EtOH, reflux °C, 20 h; (**c**) NaOH aq. 10N, reflux, 1 h.

The synthetic procedure for the preparation of the final compounds, (±)-*trans* **12a**–**17a,** is depicted in Scheme 1. The reaction of 1,3-benzodioxol-5-amine (**1**) and 3,4-dimethoxybenzenamine (**2**) with 2-bromo-propanedioic acid 1,3-diethyl ester without a solvent yielded compounds **3** and **4**, respectively. The diethyl esters **3** and **4**, and the commercially available ester, **5**, reacted with the properly substituted 3-phenyl-2-propenoic acid ethyl esters in dry ethanol and in the presence of EtONa during reflux to obtain, via a Michael intermolecular addition, a mixture of the ethyl esters diastereomer (±)-*trans* **6a**–**11a** and (±)-*cis* **6b**–**11b**. The separation of (±)-*trans* and (±)-*cis* derivatives was performed by flash chromatography. Finally, the hydrolysis of esters (±)-*trans* **6a**–**11a** with the sodium hydroxide solution 10N at reflux gave the corresponding carboxylic acid, (±)-*trans* **12a**–**17a** (Scheme 1).

**Scheme 2:**
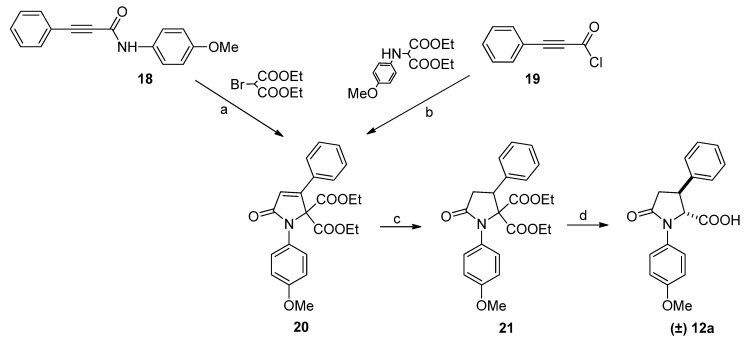
Reagents and conditions: (**a**) NaH, dry DMF, 4 days; (**b**) TEA, DMAP, dry toluene, 5 days; (**c**) H_2_/C/Pd 10%, 6 atm., 17 h; (**d**) KOH, EtOH, reflux, 4 h.

To improve its yield, 1,3-disubstituted-5-oxo-proline (±)-*trans* **12a** was also prepared following the alternative synthetic route shown in Scheme 2. The reaction of *N*-(4-methoxyphenyl)-3-phenyl-2-propynamide (**18**) with 2-bromo-propanedioic acid 1,3-diethyl ester in the presence of NaH in DMF at room temperature provided the pyrrolinone derivative (**20**) (Scheme 2, condition a) with a 16% yield. This derivative was also obtained in a better yield (49%) via a reaction of 3-phenyl-2-propynoyl chloride (**19**) and diester **5** in the presence of triethylamine (TEA) and 4-dimethylaminopyridine (DMAP) in dry toluene at room temperature (Scheme 2, condition b). Subsequently, the reduction of 1-(4-methoxyphenyl)-5-oxo-3-phenyl-2,2-pyrrolidinedicarboxylic acid 2,2-diethyl ester (**20**) was performed by hydrogenation with Pd/C under pressure to give compound **21**. Derivative **21**, in the presence of KOH at reflux, underwent hydrolysis and decarboxylation to give the final derivative, whose analytical and spectral data were consistent with those of the acid (±)-*trans* **12a**, previously described in Scheme 1.

**Scheme 3:**
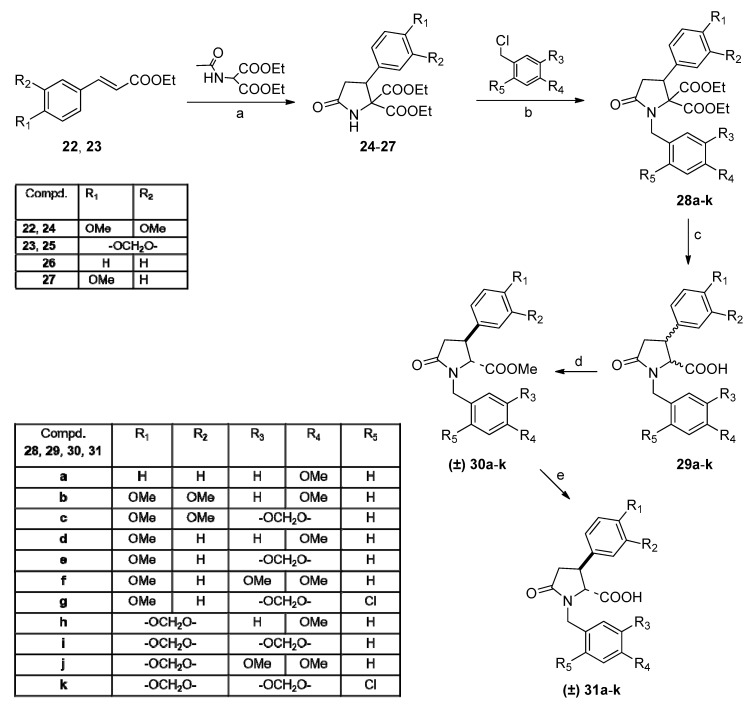
Reagents and conditions: (**a**) EtONa, dry EtOH, reflux, 12 h; (**b**) NaH, dry DMF, 1 day; (**c**) KOH, EtOH, reflux, 4 h; (**d**) SOCl_2_, MeOH, 2–4 h; (**e**) NaOH aq. 1N, MeOH, room temperature, 2–5 h.

The preparation of the benzyl derivatives **31a**–**k** is reported in Scheme 3. Derivatives **24** and **25** were obtained from the 3-phenyl-2-propenoic acid ethyl ester derivatives, **22** and **23,** and 2-(acetylamino)-propanedioic acid 1,3-diethyl ester was obtained via a Michael intermolecular addition in the presence of NaH in dry ethanol at reflux. Successively, the pyrrolidinone derivatives **24** and **25**, and the commercially available **26** and **27,** were transformed in the benzyl derivatives **28a**–**k** by a reaction with suitable 1-(chloromethyl)-substituted benzenes in the presence of NaH and dry DMF at room temperature. The benzyl diester derivatives **28a**–**k** were hydrolyzed to the corresponding acids **29a**–**k** (a mixture of *cis* and *trans*) using an ethanolic solution of potassium hydroxide at reflux. Compounds **29a**–**k** were methylated to the corresponding esters (±)-*trans* **30a**–**k** through the chloroderivatives via a reaction with thionyl chloride in methanol. The hydrolysis of the esters (±)-*trans* **30a**–**k** with sodium hydroxide at reflux produced acids (±)-*trans* **31a**–**k** in high yields.

### 2.2. Chemical Structures Elucidation

The chemical structures of the final compounds were confirmed by an ^1^H NMR spectral analysis. In particular, the identification of product configurations was made possible by an analysis of the differences of the chemical shift (δ) value and coupling constant (*J*) of the selected protons in the ^1^H NMR spectra (Table 1).

**Figure 3:**
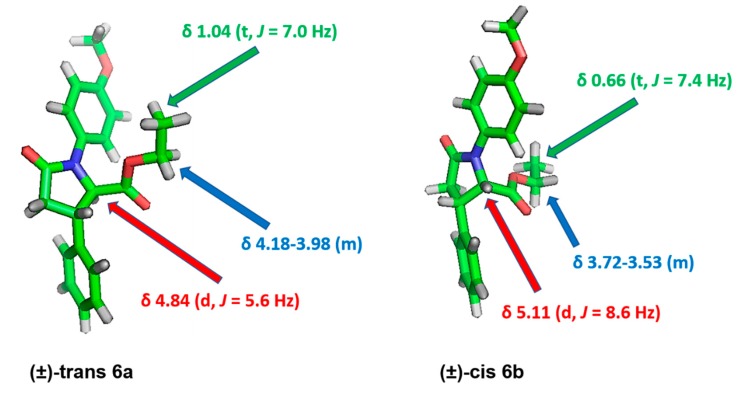
The ^1^H NMR signal attribution for the selected compounds (±)-*trans* **6a** and (±)-*cis* **6b**.

Indeed, the signals attributable to the protons of the ester function of the isomers (±)-*cis* **6b**–**11b** were present at higher fields than those of the corresponding protons of the isomers (±)-*trans* **6a–11a**, which were shielded by the anisotropy cone generated from the aryl linked at the 3-position of the oxo-proline (Figure 3). Moreover, as can be seen in Figure 3, the coupling constant of the doublet attributable to the carbon atom at the 2-position possesses a higher value in its (±)-*cis* **6b**–**11b** isomers (*J* ≅ 8.6 Hz) than that in its (±)-*trans* **6a**–**11a** (*J* ≅ 5.8 Hz).

The proton signals of the ethyl function of the ester groups at the 2-position of 1,3-disubstituted-5-oxo-pyrrolidine-dicarboxylate (**28a**–**k**) differ in their ^1^H NMR spectra and are influenced by magnetic anisotropy due to the aryl group at the 3 position of the 5-oxo-pyrrolidine-2,2-dicarboxylic acid diethyl esters. The signal of the CH_2_ protons of the *cis* isomers of the aryl, which are shielded by the anisotropy cone, gave peaks with chemical shifts at higher fields (δ 3.98–3.38). On the contrary, the signal of the CH_2_ protons of the *trans* isomers (δ 4.15–3.80) was not affected by the cone. The signal of the CH_3_ protons behaved similarly; thus, the signals of the CH_3_ protons of the *cis* isomer experience chemical shifts at higher fields (δ 0.95–0.88) than those of the CH_3_ protons of the *trans* isomer (δ 1.11–1.01). Furthermore, compounds **24** and **25,** which were not substituted at position 1, showed similar behavior regarding the proton signals of the ester functions. Therefore, we can speculate that the proton signals of the CH_2_ and CH_3_ groups of the ester functions in the *cis* isomer are influenced by the aryl group at the 3 position, while the influence of the benzyl at the 1 position is not significant. Moreover, the ^1^H NMR spectrum of compound **28g** displayed two singlets that can be assigned to the protons of the benzodioxol (-OCH_2_O-). This multiplicity could be attributed to the limited rotation of the 1-(6-chloro-1,3-benzodioxol-5-yl)methyl that, unlike other derivatives of the series, causes the presence of two atropisomeric forms that are not easily interconvertible. The configuration of (±)-*trans* **30a**–**k** was also confirmed by the differences in the chemical shift values, as well as the coupling constant values of the *cis* and *trans* isomers in the ^1^H NMR spectra. The chemical shifts (δ 3.96–3.85) and, particularly, the low values of the coupling constant (*J* = 4.4–3.0 Hz) of the doublet attributable to the protons at the 2 position of the ester function suggest a *trans* configuration for esters **30a**–**k**. These coupling constant values are in accordance with those of the ethyl ester protons of the (±)-*trans* **6a**–**11a** derivatives (*J* = 5.6–6.4 Hz) and with the literature data [31,32]. Methyl ester **30k**, analogous to what was previously observed for compound **28g**, displayed two singlets attributable to the protons of the benzodioxol in the ^1^H NMR spectrum. The chemical shifts and coupling constants (i.e., δ 4.00–3.89, *J* = 3.6–2.5 Hz) of the corresponding acids **31a**–**k** confirmed the (±)-*trans* configuration. As an example, the ^1^H NMR spectra of compounds (±)-*trans* **31a,** **31e**, **31f**, and **31g** are reported in Figures S7, S9–S12 (see Supplementary Materials).

### 2.3. Radioligand Binding Assay

Compounds (±)-*trans* **12a**–**15a** and **31a**–**k** (Table 2) were tested in binding assays on recombinant human ET_A_ and ET_B_ receptors expressed in CHO-K1 cells using [^125^I] ET-1 as the radioligand following a previously reported experimental protocol [8,33]. For the (±)*-trans* 3-(1,3-benzodioxol-5-yl)-1-[(4-methoxyphenyl)methyl]-5-oxo-proline (**31h**), the results of the binding test showed a good affinity value for the ET_A_R (*K*_i_ = 3.3 ± 1.1 µM) and no affinity for the ET_B_R. Interestingly, unlike compound **31h**, which acts as a selective ET_A_R ligand, compound **15a** did not display an affinity for either the ET_A_ or the ET_B_ receptors. Indeed, the difference in their binding properties can be attributed to a single structural difference—the presence of a methylene spacer at the 1 position on compound **31h**. Therefore, it can be assumed that the conformational freedom of the aromatic nucleus at the 1 position, due to the presence of the methylene unit, allows favorable binding of compound **31h** to the ET_A_R binding site.

The other compounds of the series, **31a**–**g** and **31i**–**k,** demonstrated specific binding lower than 50% inhibition at a 10^−5^ M concentration for ET_A_R and no affinity for ET_B_R.

Compound **12a** displayed 14% inhibition of specific binding at a 10^−5^ M concentration for ET_B_R and did not bind to the ET_A_R. Conversely, compounds **13a** and **14a** exhibited, respectively, 16% and 11% inhibition of specific binding at a 10^−5^ M concentration for the ET_A_R and no affinity for the ET_B_R.

### 2.4. Molecular Modeling Studies

**Figure 4:**
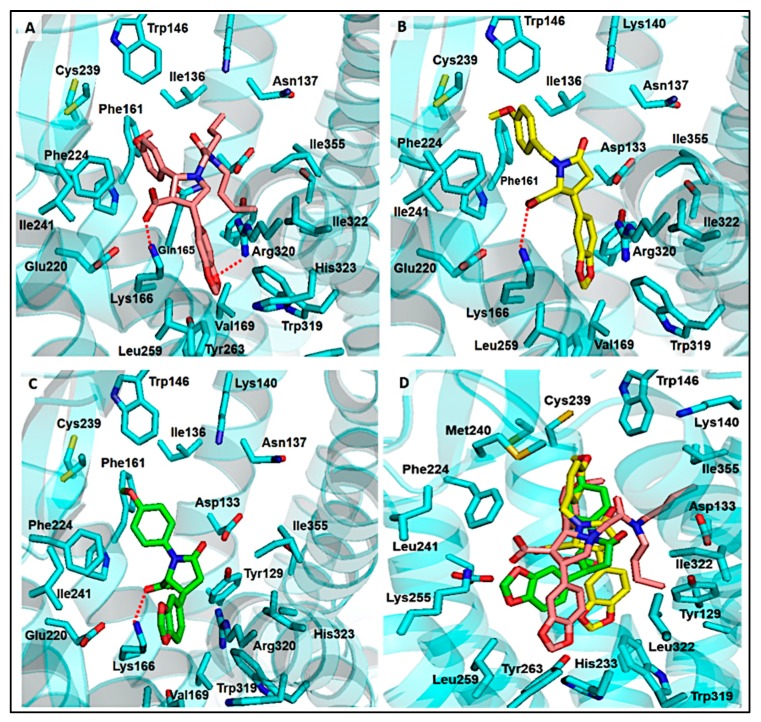
The proposed binding mode of atrasentan (**A**), **31h** (**B**), **15a** (**C**), and the overlapping ligands (**D**) within the binding site of the ET_A_R homology model. Ligands are shown as pink, yellow, and green sticks, respectively. Protein is shown as a cyan cartoon with residues within 5 Å of the ligand displayed as sticks. All hydrogen is hidden for clarity. Polar contacts are shown as red dotted lines.

A homology model of the ET_A_R was built to investigate the binding mode of the selected compounds and to gain insight into the determinants of selectivity for the ET_A_R. The recently published crystal structure of human ET_B_R was used as the template (PDB ID: 3 × 93) [34]. ET_A_ and ET_B_ receptors possess a high sequence similarity, and, most importantly, the residues constituting the putative binding site are highly conserved. Similar to ET_B_, the binding site of the ET_A_ model is wide and open towards the extracellular environment. It is mostly hydrophobic, with positively charged side chains in the central area. This large site is composed of three sub-pockets: upper (formed by Trp146, Phe224, and Cys239), lower (Tyr263 and Trp319), and eccentric (Ile136, Asn137, and Ile355). We docked atrasentan, **15a**, and **31h** into the expected binding site of ET_A_ close to the centrally located Lys166 and Arg320 (Figure 4A–D). Figure 4A shows the proposed binding mode of atrasentan. This relatively bulky compound binds vertically, extending into the upper and lower pockets. The lower methylenedioxy group is flanked by Leu259 and Trp319, while the upper methoxyphenyl moiety reaches close to Trp146 and Cys239. Further, the dibutylamino group efficiently fills the eccentric binding pocket. At the central area of the binding site, we observed the characteristic salt bridge between atrasentan and Lys166. Most of these interactions are conserved for compound **31h**, including the essential salt bridge with the same residue, Lys166 (Figure 4B). Moreover, these molecular interactions reached the upper and lower hydrophobic pockets approaching Trp146 and Trp319, respectively. In contrast, compound **15a**, due to its rigidity and lack of rotation of its methoxyphenyl group, could not interact with the Trp319 of the lower pocket (Figure 4C). Notably, we obtained other binding modes in which **15a** was able to interact with the lower pocket, but, as a consequence, it did not reach the residues of the upper pocket. The overlap in the binding poses of atrasentan, **15a**, and **31h** (Figure 4D) clearly show that **31h** can adopt an extended orientation similar to atrasentan, filling the upper and lower hydrophobic pockets, while **15a**, due to its rigid curved structure, might not interact with the same residues. It is worth noting that previous biochemical experiments and SAR studies of other ET antagonists, such as bosentan, highlighted the importance of interactions with the aforementioned upper and lower hydrophobic pockets, especially Trp319 in the middle of helix 6 [34]. This fact could explain, at least in part, the significantly lower activity of **15a** compared to **31h** and atrasentan, which makes hydrophobic contacts favorable with this residue.

## 3. Materials and Methods

### 3.1. Chemistry

Melting points were determined in a Gallenkamp apparatus (London, UK) with a digital thermometer MFB-595 in glass capillary tubes and were uncorrected. The elemental analyses for C, H, and N were within ± 0.4% of the theoretical values and were performed on a Carlo Erba Elemental Analyzer Mod 1108 apparatus (Carlo Erba Reagents S.r.l, Milan, IT). The IR spectra were recorded in KBr disks (solid samples) or NaCl plates (oil samples) on a Perkin Elmer 1600 Series FT-IR spectrometer (PerkinElmer (UK) Holdings Ltd., Beaconsfield, UK). The ^1^H NMR and ^13^C NMR spectra were recorded on a Varian Inova Unity 200 and a Varian Inova Unity 500 spectrometer (Agilent, Santa Clara, CA, USA), respectively, using a CDCl_3_ or DMSO-*d*_6_ solution. The chemical shifts are given in δ values (ppm), using tetramethylsilane as the internal standard; the coupling constants (*J*) are given in hertz (Hz). The signal multiplicities are characterized as s (singlet), d (doublet), dd (double doublet), t (triplet), q (quartet), m (multiplet), or br (broad signal). All the synthesized compounds were tested for purity on TLC (an aluminum sheet coated with silica gel 60 F254, Merck, Darmstadt, DE) and visualized by UV (λ = 254 and 366 nm). All chemicals and solvents were of reagent grade and purchased from commercial vendors (Merck KGaA, Darmstadt, DE). The synthetic procedures and experimental data of the intermediate compounds **3**, **4**, (±) **6a**–**11a**, (±) **6b**–**11b**, **20**, **21**, **24**, **25**, (±) **28a**–**k**, (±) **29a**–**k**, and (±) **30a**–**k** are reported in the Supplementary Materials.

#### 3.1.1. General Procedure for the Synthesis of (±)-Trans 1,3-substituted-5-oxo-prolines **12a** and **15a**

An aqueous solution of NaOH 10N (0.37 mL, 3.75 mmol) was added to a suspension of ester (±)-*trans* **6a** or **9a** (0.20 mmol) in ethanol (0.4 mL). This mixture was kept at reflux for 1 h. After being cooled, the mixture was diluted with water (20 mL). We then used acidification with concentrated HClto obtain a solid, which was filtered, washed with water, and dried. Using this procedure, the following compounds were obtained:

*(±)-trans 1-(4-Methoxyphenyl)-5-oxo-3-phenyl-proline* (**12a**): Compound **12a** was obtained pure (87%), mp 198–200 °C. IR (KBr, cm^−1^, selected lines): 2915, 1739, 1648, 1514, 1184, 1032, 818, 636.^1^H NMR (DMSO-*d*_6_): δ 13.19 (br s, 1H, COOH exchanges with D_2_O), 7.57–7.20 (m, 5H + 2H, Ar), 7.02–6.87 (m, 2H, Ar), 4.71 (d, *J* = 5.0 Hz, 1H, CHCOO), 3.73 (s, 3H, OCH_3_), 3.72–3.58 (m, 1H, CHAr), 3.00 (dd, ^3^*J* = 8.8 Hz; ^2^*J* = 16.7 Hz, 1H, C*H*_A_H_B_CHAr), 2.56 (dd, ^3^*J* = 6.0 Hz; ^2^*J* = 16.7 Hz, 1H, CH_A_*H*_B_CHAr). Anal. Calcd. for (C_18_H_17_NO_4_): C, 69.44; H, 5.50; N, 4.50. Found: C, 69.60; H, 5.39; N, 4.37.

(±)-*trans* 3-(1,3-Benzodioxol-5-yl)-1-(4-methoxyphenyl)-5-oxo-proline (**15a**): Compound **15a** was obtained pure (91%), mp 200–202 °C. IR (KBr, cm^−1^, selected lines): 2913, 1739, 1647, 1513, 1249, 1035, 823, 644. ^1^H NMR (DMSO-*d*_6_): δ 7.43–7.32 (m, 2H, Ar), 7.08–6.77 (m, 2H + 3H, Ar), 6.01 (s, 2H, OCH_2_O), 4.69 (d, *J* = 5.6 Hz, 1H, CHCOO), 3.74 (s, 3H, OCH_3_), 3.64–3.50 (m, 1H, CHAr), 2.93 (dd, ^3^*J* = 9.4 Hz; ^2^*J* = 17.0 Hz, 1H, C*H_A_*H_B_CHAr), 2.55 (dd, ^3^*J* = 6.6 Hz; ^2^*J* = 17.0 Hz, 1H, CH_A_*H_B_*CHAr). Anal. Calcd. for (C_19_H_17_NO_6_): C, 64.22; H, 4.82; N, 3.94. Found: C, 64.08; H, 4.56; N, 4.06.

#### 3.1.2. General Procedure for the Synthesis of (±)-trans 1,3-Subtituted-5-oxo-prolines (**13a**, **14a**, **16a**, **17a**)

An aqueous solution of NaOH 10N (0.5 mL, 5.00 mmol) was added to a suspension of appropriate (±)-*trans* ester (**7a**, **8a**, **10a**, **11a)** (0.20 mmol) in ethanol (2 mL). This mixture was kept at reflux for 1 h. After being cooled, the mixture was diluted with water (20 mL) and extracted with ethyl acetate. The water layer was collected, neutralized with concentrated HCl, and extracted with ethyl acetate (2 × 20 mL). The latter organic layer was dried over anhydrous sodium sulfate and evaporated to dryness under reduced pressure to obtain an oily crude product, which was purified by flash column chromatography using ethyl acetate/methanol (7:3, *v*:*v*) as an eluent. Using evaporation of the solvent of homogeneous fractions compounds, (±)-*trans* **13a**, **14a**, **16a**, **17a** were obtained as sodium salts, and then the salts were transformed in the corresponding acids by acidification of their aqueous solution with 1N HCl. Using this procedure, the following compounds were obtained:

##### (±)-trans 1-(1,3-Benzodioxol-5-yl)-3-(4-methoxyphenyl)-5-oxo-proline (**13a**)

Sodium salt (±)-*trans* **13a** was obtained pure (88%); mp 232 °C. IR (KBr, cm^−1^, selected lines): 2961, 1612, 1508, 1250, 1181, 1036, 809, 527. ^1^H NMR (DMSO-*d*_6_): δ 7.28–7.12 (m, 3H, Ar), 6.94–6.80 (m, 2H + 2H, Ar), 5.98 (s, 2H, OCH_2_O), 4.14 (d, *J* = 4.0 Hz, 1H, CHCOO), 3.71 (s, 3H, OCH_3_), 3.58–3.22 (m, 1H, CHAr), 3.00 (dd, ^3^*J* = 9.2 Hz; ^2^*J* = 16.8 Hz, 1H, C*H*_A_H_B_CHAr), 2.32 (dd, ^3^*J* = 2.8 Hz; ^2^*J* = 16.8 Hz, 1H, CH_A_*H*_B_CHAr). Anal. Calcd. for (C_19_H_16_NaNO_6_): C, 60.48; H, 4.27; N, 3.71. Found: C, 60.62; H, 4.35; N, 3.82.

Acid (±)-*trans* **13a**. ^1^H NMR (DMSO-*d*_6_): δ 7.33–7.24 (m, 2H, Ar), 7.17–7.12 (m, 1H, Ar), 6.97–6.87 (m, 2H + 1H, Ar), 6.83–6.74 (m, 1H, Ar), 6.02 (s, 2H, OCH_2_O), 4.67 (d, *J* = 5.2 Hz, 1H, CHCOO), 3.73 (s, 3H, OCH_3_), 3.65–3.52 (m, 1H, CHAr), 2.95 (dd, ^3^*J* = 9.0 Hz; ^2^*J* = 18.0 Hz, 1H, C*H*_A_H_B_CHAr), 2.54 (dd, ^3^*J* = 6.8 Hz, ^2^*J* = 18.0 Hz, 1H, CH_A_*H*_B_CHAr). Anal. Calcd. for (C_19_H_17_NO_6_): C, 64.22; H, 4.82; N, 3.94. Found: C, 64.34; H, 4.61; N, 3.83.

##### (±)-trans 1,3-Di(1,3-benzodioxol-5-yl)-5-oxo-proline (**14a**)

Sodium salt (±)-*trans* **14a** was obtained pure (88%); mp 214 °C. IR (KBr, cm^−1^, selected lines): 3379, 2898, 1693, 1504, 1246, 1038, 933, 810. ^1^H NMR (DMSO-*d*_6_): δ 7.26–7.20 (m, 1H, Ar), 6.94–6.80 (m, 2H + 2H, Ar), 6.76–6.67 (m, 1H, Ar), 5.98 (s, 2H + 2H, OCH_2_O), 4.25 (d, *J* = 3.0 Hz, 1H, CHCOO), 3.60–3.21 (m, 1H, CHAr), 2.96 (dd, ^3^*J* = 8.6 Hz; ^2^*J* = 16.6 Hz, 1H, C*H*_A_H_B_CHAr), 2.36 (dd, ^3^*J* = 4.2 Hz; ^2^*J* = 16.6 Hz, 1H, CH_A_*H*_B_CHAr). Anal. Calcd. for (C_19_H_14_NaNO_7_): C, 58.32; H, 3.61; N, 3.58. Found: C, 58.19; H, 3.50; N, 3.65.

Acid (±)-*trans* **14a**; mp>250 °C dec. IR (KBr, cm^−1^, selected lines): 2957, 1649, 1488, 1268, 1103, 1037, 935, 802. ^1^H NMR (DMSO-*d*_6_): δ 7.16–7.11 (m, 1H, Ar), 7.05–7.00 (m, 1H, Ar), 6.95–6.76 (m, 2H + 2H, Ar), 6.02 (s, 2H, OCH_2_O), 6.01 (s, 2H, OCH_2_O), 4.71 (d, *J* = 5.6 Hz, 1H, CHCOO), 3.64–3.50 (m, 1H, CHAr), 2.92 (dd, ^3^*J* = 8.8 Hz; ^2^*J* = 16.8 Hz, 1H, C*H*_A_H_B_CHAr), 2.55 (dd, ^3^*J* = 7.4 Hz; ^2^*J* = 16.8 Hz, 1H, CH_A_*H*_B_CHAr). Anal. Calcd. for (C_19_H_15_NO_7_): C, 61.79; H, 4.09; N, 3.79. Found: C, 61.60; H, 4.00; N, 3.85.

##### (±)-trans 3-(3,4-Dimethoxyphenyl)-1-(4-methoxyphenyl)-5-oxo-proline (**16a**)

Sodium salt (±)-*trans* **16a** was obtained pure (80%); mp 224 °C. IR (KBr, cm^−1^, selected lines): 2937, 1682, 1516, 1401, 1249, 1025, 829, 520. ^1^H NMR (DMSO-*d*_6_): δ 7.52–7.42 (m, 2H, Ar), 6.92–6.81 (m, 2H + 2H, Ar), 6.77–6.69 (m, 1H, Ar), 4.16 (d, *J* = 2.4 Hz, 1H, CHCOO), 3.70 (s, 3H + 3H + 3H, OCH_3_), 3.50–3.23 (m, 1H, CHAr), 2.99 (dd, ^3^*J* = 8.8 Hz; ^2^*J* = 16.6 Hz, 1H, C*H*_A_H_B_CHAr), 2.32 (dd, ^3^*J* = 3.0 Hz; ^2^*J* = 16.6 Hz, 1H, CH_A_*H*_B_CHAr). Anal. Calcd. for (C_20_H_20_NaNO_6_): C, 61.07; H, 5.12; N, 3.56. Found: C, 60.98; H, 5.26; N, 3.50.

Acid (±)-*trans* **16a**; mp 216–220 °C. IR (KBr, cm^−1^, selected lines): 2963, 1649, 1517, 1260, 1025, 800, 678, 517. ^1^H NMR (DMSO-*d*_6_): δ 13.1 (br s, 1H, COOH which exchanges with D_2_O), 7.42–7.32 (m, 2H, Ar), 7.10–6.82 (m, 2H + 3H, Ar), 4.71 (d, *J* = 5.4 Hz, 1H, CHCOO), 3.76 (s, 3H, OCH_3_), 3.73 (s, 3H + 3H, OCH_3_), 3.65–3.50 (m, 1H, CHAr), 2.94 (dd, ^3^*J* = 8.8 Hz; ^2^*J* = 17.0 Hz, 1H, C*H*_A_H_B_CHAr), 2.60 (dd, ^3^*J* = 6.6 Hz; ^2^*J* = 17.0 Hz, 1H, CH_A_*H*_B_CHAr). Anal. Calcd. for (C_20_H_21_NO_6_): C, 64.68; H, 5.70; N, 3.77. Found: C, 64.75; H, 5.61; N, 3.70.

##### (±)-trans 1-(3,4-Dimethoxyphenyl)-3-(4-methoxyphenyl)-5-oxo-proline (**17a**)

Sodium salt (±)-*trans* **17a** was obtained pure (51%); mp > 250 °C dec. IR (KBr, cm^−1^, selected lines): 2961, 1682, 1515, 1402, 1255, 1025, 802, 536. ^1^H NMR (DMSO-*d*_6_): δ 7.49–7.42 (m, 1H, Ar), 7.21–7.10 (m, 2H, Ar), 7.01–6.80 (m, 2H + 2H, Ar), 4.13 (d, *J* = 3.0 Hz, 1H, CHCOO), 3.71 (s, 3H, OCH_3_), 3.70 (s, 3H + 3H, OCH_3_), 3.50–3.25 (m, 1H, CHAr), 3.02 (dd, ^3^*J* = 8.6 Hz; ^2^*J* = 16.6 Hz, 1H, C*H*_A_H_B_CHAr), 2.30 (dd, ^3^*J* = 3.0 Hz; ^2^*J* = 16.6 Hz, 1H, CH_A_*H*_B_CHAr). Anal. Calcd. for (C_20_H_20_NaNO_6_): C, 61.07; H, 5.12; N, 3.56. Found: C, 61.20; H, 5.22; N, 3.69.

Acid (±)-*trans* **17a**. ^1^H NMR (DMSO-*d*_6_): δ 7.35–7.20 (m, 3H, Ar), 6.99–6.82 (m, 2H + 2H, Ar), 4.70 (d, *J* = 5.4 Hz, 1H, CHCOO), 3.74 (s, 3H, OCH_3_), 3.73 (s, 3H, OCH_3_), 3.72 (s, 3H, OCH_3_), 3.66–3.53 (m, 1H, CHAr), 2.97 (dd, ^3^*J* = 9.0 Hz; ^2^*J* = 17.0 Hz, 1H, C*H*_A_H_B_CHAr), 2.56 (dd, ^3^*J* = 6.2 Hz; ^2^*J* = 17.0 Hz, 1H, CH_A_*H*_B_CHAr). Anal. Calcd. for (C_20_H_21_NO_6_): C, 64.68; H, 5.70; N, 3.77. Found: C, 64.60; H, 5.78; N, 3.55.

#### 3.1.3. (±)-Trans 1-(4-Methoxyphenyl)-5-oxo-3-phenyl-proline (**12a**)

An aqueous solution of KOH 1N (0.3 mL, 0.3 mmol) was added to a suspension of diester **21** (0.050 g, 0.12 mmol) in ethanol (0.7 mL). The mixture was kept at reflux for 4 h. After being cooled, the solvent was evaporated to dryness under reduced pressure. The crude product was solubilized with ethyl acetate (10 mL), washed with water (2 × 10 mL), and acidified with concentrated HCl. The organic layer was then collected and dried over anhydrous sodium sulfate. After evaporation of the solvent under reduced pressure, we obtained compound (±)-*trans* **12a** (0.020 g, 53%), which possesses the same analytical and spectral data of the compound obtained by hydrolysis of the ester (±)-*trans* **6a**.

#### 3.1.4. General Procedure for the Synthesis of (±)-Trans 1,3-disubstituted-5-oxo-proline **31a**–**k**

An aqueous solution of NaOH 1N (0.77 mL, 0.77 mmol) was added to a solution of the appropriate methyl ester (**30a**–**k**) (0.29 mmol) in methanol (4.0 mL). This mixture was stirred at room temperature for 2–5 h. Then, the solvent was removed at reduced pressure, water was added (2 mL), and the solution was extracted with ethyl acetate (2 mL). The water layer was then collected, acidified with concentrated HCl, and extracted with ethyl acetate (3 × 5 mL). The organic layer of the latter extraction was collected and dried over anhydrous sodium sulfate. By evaporating the solvent to dryness, we obtained the desired product. The following compounds were obtained with this procedure.

*(±)-trans 1-[(Methoxyphenyl)methyl]-3-phenyl-5-oxo-proline* (**31a**): The title compound was obtained as a pure solid (94%); mp 105–108 °C. IR (KBr, cm^−1^, selected lines): 2944, 1737, 1512, 1246, 1178, 1031, 841, 764. ^1^H NMR (CDCl_3_): δ 10.21 (br s, 1H, COOH exchanges with D_2_O), 7.32–7.18 (m, 3H, Ar), 7.18–7.00 (m, 2H + 2H, Ar), 6.83–6.70 (m, 2H, Ar), 5.14 (d, *J* = 14.8 Hz, 1H, NC*H*_A_H_B_), 3.97 (d, *J* = 14.8 Hz, 1H, NCH_A_*H*_B_), 3.97 (d, *J* = 3.2 Hz, 1H C*H*COOH), 3.74 (s, 3H, OCH_3_), 3.67–3.53 (m, 1H, CHAr), 3.11 (dd, ^3^*J* = 9.2 Hz, ^2^*J* = 17.6 Hz, 1H, COC*H*_A_H_B_), 2.62 (dd, ^3^*J* = 3.8 Hz, ^2^*J* = 17.6 Hz, 1H, COCH_A_*H*_B_). ^13^C NMR (CDCl_3_): δ 175.34, 173.63, 159.29, 142.18, 130.06, 128.94, 127.44, 126.82, 126.32, 114.08, 66.17, 55.18, 45.31, 41.03, 38.04. Anal. Calcd. for (C_19_H_19_NO_4_): C, 70.14; H, 5.89; N, 4.31. Found: C, 69.98; H, 5.79; N, 4.22.

*(±)-trans 3-(3,4-Dimethoxyphenyl)-1-[(4-methoxyphenyl)methyl]-5-oxo-proline* (**31b**): The title compound was obtained as a pure solid (92%); mp 144–147 °C. IR (KBr, cm^−1^, selected lines): 2934, 1734, 1640, 1514, 1247, 1026, 814, 759. ^1^H NMR (CDCl_3_): δ 11.09 (br s, 1H, COOH exchanges with D_2_O), 7.20–7.05 (m, 2H, Ar), 6.82–6.50 (m, 2H + 3H, Ar), 5.17 (d, *J* = 14.6 Hz, 1H, NC*H*_A_H_B_), 4.00 (d, *J* = 2.8 Hz, 1H, C*H*COOH), 3.95 (d, *J* = 14.6 Hz, 1H, NCH_A_*H*_B_), 3.82 (s, 3H, OCH_3_), 3.74 (s, 3H, OCH_3_), 3.73 (s, 3H, OCH_3_), 3.68–3.50 (m, 1H, CHAr), 3.14 (dd, ^3^*J* = 9.2 Hz, ^2^*J* = 17.1 Hz, 1H, COC*H*_A_H_B_), 2.65 (dd, ^3^*J* = 3.6 Hz, ^2^*J* = 17.1 Hz, 1H, COCH_A_*H*_B_). Anal. Calcd. for (C_21_H_23_NO_6_): C, 65.44; H, 6.02; N, 3.63. Found: C, 65.34; H, 6.18; N, 3.74.

*(±)-trans 1-[(1,3-Benzodioxol-5-yl)methyl]-3-(3,4-dimethoxyphenyl)-5-oxo-proline* (**31c**): The title compound was obtained as a pure oil (89%). IR (NaCl, cm^−1^, selected lines): 2935, 1736, 1641, 1446, 1244, 1032, 873, 810. ^1^H NMR (CDCl_3_): δ 9.27 (br s, 1H, COOH exchanges with D_2_O), 6.81–6.60 (m, 2H + 3H, Ar), 6.60–6.48 (m, 1H, Ar), 5.89 (s, 2H, OCH_2_O), 5.14 (d, *J* = 14.6 Hz, 1H, NC*H*_A_H_B_), 3.99 (d, *J* = 2.8 Hz, 1H, C*H*COOH), 3.96 (d, *J* = 14.6 Hz, 1H, NCH_A_*H*_B_), 3.83 (s, 3H, OCH_3_), 3.76 (s, 3H, OCH_3_), 3.65–3.50 (m, 1H, CHAr), 3.12 (dd, ^3^*J* = 9.4 Hz, ^2^*J* = 17.6 Hz, 1H, COC*H*_A_H_B_), 2.63 (dd, ^3^*J* = 3.2 Hz, ^2^*J* = 17.6 Hz, 1H, COCH_A_*H*_B_). Anal. Calcd. for (C_21_H_21_NO_7_): C, 63.15; H, 5.30; N, 3.51. Found: C, 63.27; H, 5.39; N, 3.39.

*(±)-trans 1-[(4-Methoxyphenyl)methyl]-3-(4-methoxyphenyl)-5-oxo-proline* (**31d**): The title compound was obtained as a pure solid (93%); mp 107–110 °C. IR (KBr, cm^−1^, selected lines): 2934, 1735, 1641, 1458, 1250, 1032, 830, 732. ^1^H NMR (CDCl_3_): δ 7.20–7.08 (m, 2H, Ar), 7.04–6.94 (m, 2H, Ar), 6.84–6.70 (m, 4H, Ar), 5.13 (d, *J* = 14.7 Hz, 1H, NC*H*_A_H_B_), 3.93 (d, *J* = 14.7 Hz, 1H, NCH_A_*H*_B_), 3.89 (d, *J* = 3.2 Hz, 1H, C*H*COOH), 3.75 (s, 3H + 3H, OCH_3_), 3.60–3.45 (m, 1H, CHAr), 3.05 (dd, ^3^*J* = 9.6 Hz, ^2^*J* = 17.4 Hz, 1H, COC*H*_A_H_B_), 2.54 (dd, ^3^*J* = 4.0 Hz, ^2^*J* = 17.4 Hz, 1H, COCH_A_*H*_B_). Anal. Calcd. for (C_20_H_21_NO_5_): C, 67.59; H, 5.96; N, 3.94. Found: C, 67.40; H, 5.87; N, 4.07.

*(±)-trans 1-[(1,3-Benzodioxol-5-yl)methyl]-3-(4-methoxyphenyl)-5-oxo-proline* (**31e**): The title compound was obtained as a pure solid (83%); mp 115–117 °C. IR (KBr, cm^−1^, selected lines): 2929, 1735, 1642, 1444, 1249, 1036, 926, 827. ^1^H NMR (CDCl_3_): δ 7.05–6.98 (m, 2H, Ar), 6.83–6.76 (m, 2H, Ar), 6.72–6.65 (m, 3H, Ar), 5.91 (s, 2H, OCH_2_O), 5.08 (d, *J* = 14.2 Hz, 1H, NC*H*_A_H_B_), 4.64 (br s, 1H, COOH exchanges with D_2_O), 3.92 (d, *J* = 3.6 Hz, 1H, C*H*COOH), 3.91 (d, *J* = 14.2 Hz, 1H, NCH_A_*H*_B_), 3.77 (s, 3H, OCH_3_), 3.61–3.50 (m, 1H, CHAr), 3.05 (dd, ^3^*J* = 9.0 Hz, ^2^*J* = 17.2 Hz, 1H, COC*H*_A_H_B_), 2.57 (dd, ^3^*J* = 3.8 Hz, ^2^*J* = 17.2 Hz, 1H, COCH_A_*H*_B_). Anal. Calcd. for (C_20_H_19_NO_6_): C, 65.03; H, 5.18; N, 3.79. Found: C, 64.89; H, 5.07; N, 3.67.

*(±)-trans 1-[(3,4-Dimethoxyphenyl)methyl]-3-(4-methoxyphenyl)-5-oxo-proline* (**31f**): The title compound was obtained as a pure solid (99%); mp 171–172 °C. IR (KBr, cm^−1^, selected lines): 2922, 1722, 1628, 1457, 1264, 1150, 1027, 874. ^1^H NMR (CDCl_3_): δ 7.07–6.98 (m, 2H, Ar), 6.83–6.67 (m, 2H + 3H, Ar), 6.09 (br s, 1H, COOH exchanges with D_2_O), 5.12 (d, *J* = 14.6 Hz, 1H, NC*H*_A_H_B_), 3.97 (d, *J* = 14.6 Hz, 1H, NCH_A_*H*_B_), 3.93 (d, *J* = 2.6 Hz, 1H, C*H*COOH), 3.83 (s, 3H, OCH_3_), 3.77 (s, 3H, OCH_3_), 3.76 (s, 3H, OCH_3_), 3.63–3.52 (m, 1H, CHAr), 3.08 (dd, ^3^*J* = 8.8 Hz, ^2^*J* = 17.6 Hz, 1H, COC*H*_A_H_B_), 2.59 (dd, ^3^*J* = 3.6 Hz, ^2^*J* = 17.6 Hz, 1H, COCH_A_*H*_B_). Anal. Calcd. for (C_21_H_23_NO_6_): C, 65.44; H, 6.02; N, 3.63. Found: C, 65.26; H, 6.23; N, 3.69.

*(±)-trans 1-[(6-Chloro-1,3-benzodioxol-5-yl)methyl]-3-(4-methoxyphenyl)-5-oxo-proline* (**31g**): The title compound was obtained as a pure solid (79%); mp 154–156 °C. IR (KBr, cm^−1^, selected lines): 2908, 1724, 1630, 1481, 1258, 1034, 931, 822. ^1^H NMR (CDCl_3_): δ 7.09–7.02 (m, 2H, Ar), 6.90–6.65 (m, 2H + 2H, Ar), 5.94 (s, 2H, OCH_2_O), 5.06 (d, *J* = 14.7 Hz, 1H, NC*H*_A_H_B_), 4.22 (d, *J* = 14.7 Hz, 1H, NCH_A_*H*_B_), 3.94 (d, *J* = 3.4 Hz, 1H, C*H*COOH), 3.78 (s, 3H, OCH_3_), 3.60–3.48 (m, 1H, CHAr), 3.02 (dd, ^3^*J* = 9.4 Hz, ^2^*J* = 17.1 Hz, 1H, COC*H*_A_H_B_), 2.56 (dd, ^3^*J* = 4.0 Hz, ^2^*J* = 17.1 Hz, 1H, COCH_A_*H*_B_). Anal. Calcd. for (C_20_H_18_ClNO_6_): C, 59.49; H, 4.49; N, 3.47. Found: C, 59.61; H, 4.62; N, 3.56.

*(±)-trans 3-(1,3-Benzodioxol-5-yl)-1-[(4-methoxyphenyl)methyl]-5-oxo-proline* (**31h**): The title compound was obtained as a pure solid (78%); mp 113–115 °C. IR (KBr, cm^−1^, selected lines): 2931, 1735, 1640, 1448, 1247, 1036, 939, 815. ^1^H NMR (CDCl_3_): δ 9.15 (br s, 1H, COOH exchanges with D_2_O), 7.20–7.08 (m, 2H, Ar), 6.85–6.75 (m, 2H, Ar), 6.75–6.60 (m, 1H, Ar), 6.60–6.45 (m, 2H, Ar), 5.89 (s, 2H, OCH_2_O), 5.13 (d, *J* = 14.5 Hz, 1H, NC*H*_A_H_B_), 3.95 (d, *J* = 14.5 Hz, 1H, NCH_A_*H*_B_), 3.90 (d, *J* = 3.4 Hz, 1H, C*H*COOH), 3.75 (s, 3H, OCH_3_), 3.58–3.46 (m, 1H, CHAr), 3.08 (dd, ^3^*J* = 9.2 Hz, ^2^*J* = 17.5 Hz, 1H, COC*H*_A_H_B_), 2.56 (dd, ^3^*J* = 3.8 Hz, ^2^*J* = 17.5 Hz, 1H, COCH_A_*H*_B_). Anal. Calcd. for (C_20_H_19_NO_6_): C, 65.03; H, 5.18; N, 3.79. Found: C, 64.87; H, 5.08; N, 3.66.

*(±)-trans 1-[(1,3-Benzodioxol-5-yl)methyl]-3-(1,3-benzodioxol-5-yl)-5-oxo-proline* (**31i**): The title compound was obtained as a pure solid (65%); mp 155–157 °C. IR (KBr, cm^−1^, selected lines): 2904, 1716, 1614, 1247, 1040, 924, 821, 770. ^1^H NMR (CDCl_3_): δ 6.78–6.62 (m, 4H, Ar), 6.60–6.55 (m, 2H, Ar), 5.93 (s, 2H + 2H, OCH_2_O), 5.08 (d, *J* = 14.6 Hz, 1H, NC*H*_A_H_B_), 3.91 (d, *J* = 3.4 Hz, 1H, C*H*COOH), 3.91 (d, *J* = 14.6 Hz, 1H, NCH_A_*H*_B_), 3.55–3.45 (m, 1H, CHAr), 3.04 (dd, ^3^*J* = 9.2 Hz, ^2^*J* = 17.6 Hz, 1H, COC*H*_A_H_B_), 2.53 (dd, ^3^*J* = 4.0 Hz, ^2^*J* = 17.6 Hz, 1H, COCH_A_*H*_B_). Anal. Calcd. for (C_20_H_17_NO_7_): C, 62.66; H, 4.47; N, 3.65. Found: C, 62.51; H, 4.29; N, 3.55.

*(±)-trans 3-(1,3-Benzodioxol-5-yl)-1-[(3,4-dimethoxyphenyl)methyl]-5-oxo-proline* (**31j**): The title compound was obtained as a pure solid (86%); mp 131–132 °C. IR (KBr, cm^−1^, selected lines): 2934, 1736, 1643, 1447, 1030, 928, 811, 668. ^1^H NMR (CDCl_3_): δ 6.94 (br s, 1H, COOH exchanges with D_2_O), 6.80–6.70 (m, 3H, Ar), 6.70–6.62 (m, 1H, Ar), 6.62–6.52 (m, 2H, Ar), 5.91 (s, 2H, OCH_2_O), 5.14 (d, *J* = 14.6 Hz, 1H, NC*H*_A_H_B_), 3.95 (d, *J* = 14.6 Hz, 1H, NCH_A_*H*_B_), 3.90 (d, *J* = 2.8 Hz, 1H, C*H*COOH), 3.83 (s, 3H, OCH_3_), 3.80 (s, 3H, OCH_3_), 3.60–3.50 (m, 1H, CHAr), 3.08 (dd, ^3^*J* = 9.6 Hz, ^2^*J* = 17.5 Hz, 1H, COC*H*_A_H_B_), 2.56 (dd, ^3^*J* = 3.2 Hz, ^2^*J* = 17.5 Hz, 1H, COCH_A_*H*_B_). Anal. Calcd. for (C_21_H_21_NO_7_): C, 63.15; H, 5.30; N, 3.51. Found: C, 62.86; H, 5.11; N, 3.80.

*(±)-trans 3-(1,3-Benzodioxol-5-yl)-1-[(6-chloro-1,3-benzodioxol-5-yl)methyl]-5-oxo-proline* (**31k**): The title compound was obtained as a pure solid (77%); mp 126–128 °C. IR (KBr, cm^−1^, selected lines): 2908, 1735, 1489, 1242, 1037, 930, 807, 664. ^1^H NMR (CDCl_3_): δ 8.15 (br s, 1H, COOH exchanges with D_2_O), 6.80–6.75 (m, 2H, Ar), 6.70–6.66 (m, 1H, Ar), 6.61–6.56 (m, 2H, Ar), 5.92 (s, 2H, OCH_2_O), 5.90 (s, 2H, OCH_2_O), 5.06 (d, *J* = 15.0 Hz, 1H, NC*H*_A_H_B_), 4.20 (d, *J* = 15.0 Hz, 1H, NCH_A_*H*_B_), 3.91 (d, *J* = 2.5 Hz, 1H, C*H*COOH), 3.55–3.49 (m, 1H, CHAr), 3.03 (dd, ^3^*J* = 9.5 Hz, ^2^*J* = 17.5 Hz, 1H, COC*H*_A_H_B_), 2.55 (dd, ^3^*J* = 3.5 Hz, ^2^*J* = 17.5 Hz, 1H, COCH_A_*H*_B_). Anal. Calcd. for (C_20_H_16_ClNO_7_): C, 57.50; H, 3.86; N, 3.35. Found: C, 57.64; H, 3.97; N, 3.44.

### 3.2. Radioligand Binding Assay

The binding was determined using human recombinant ET_A_ or ET_B_R (CHO-K1 cell line, Euroscreen). The cells were resuspended in Tris HCl, 50 mM, pH 7.5, containing 10 mM MgCl_2_ and used at a concentration of 0.08 μg per sample for ET_A_R and 0.7 μg per sample for ET_B_R. The assay [33] was initiated by adding 25 μL of [^125^I]endothelin-1 (0.2–0.4 nM; specific activity 2000 Ci mmol^−1^, Amersham) in 0.05% protease-free BSA in a final volume of 100 μL (tubes minisorp, Nunc). Non-specific binding was obtained in the presence of 1 μM endothelin-1 and 1 μM BQ-123 for ET_A_R or 1 μM endothelin-1 and 1 μM BQ-788 for ET_B_R. The compounds were dissolved in DMSO and tested at 10^−5^ and 10^−7^ M in triplicate, and the inhibition curves of endothelin-1, BQ-123, and BQ-788 were obtained using five to seven different concentrations in triplicate. The incubation (30 °C, 60 min) was stopped by dilution with a cold buffer (Tris HCl, 20 mM, pH 7.5 containing 10 mM MgCl_2_) and filtration through GF/C filters presoaked in 0.1% protease-free BSA. The filters were washed three times with the same buffer using a Brandel cell harvester and were counted in a gamma counter with 90% efficiency. The inhibition curves were analyzed using the “Allfit” [35] program, and the *K*_i_ values were derived from the IC_50_ values using the Cheng and Prusoff [36] equation.

### 3.3. Molecular Modeling Studies

The homology model of endothelin ET_A_R was developed based on the recently published crystal structure of human ET_B_R in a complex with the antagonist K-8794 (PDB ID: 3 × 93) at 2.2 Å. The sequence of human ET_A_R was retrieved from the Uniprot database (Accession number: P25101). Sequences of endothelin ET_A_R and ET_B_R were aligned using the Clustal Omega online tool and corrected using BioEdit. The homology modeling step was performed using Modeller v9.20. This software uses sequence alignment to extract a large number of spatial restraints for homology modeling of the target protein. These spatial restraints are then expressed as PDFs, which are combined into an objective function optimized by conjugate gradients and MD-simulated annealing. Ten loop-refined models were generated. The model with the lowest PDF violations was selected for the next steps. All hydrogen atoms were added to the model using the Molecular Operating Environment software (MOE), v2016. Next, the *N*-termini of the 7 TM helices were capped with ACE, while the C-termini were capped with NMA groups. The side chains of the Asn and Gln residues were reoriented to optimize the H-bonding network. The crude model was minimized in MOE. First, all heavy atoms were kept fixed, and the hydrogen atoms were allowed to optimize their positions. Second, the entire backbone of the protein was kept fixed, and the side chains were minimized using the same parameters. Finally, the whole molecule including the backbone was tethered with gradually reducing positional restraints. The three minimization steps consisted of 6000 steps each, in which the first 1000 steps involved the steepest descents, and the last 5000 were L-BFGS, with gradually reducing positional restraints of 100, 50, 25 10, and 5 kcal/mol/Å2. The final homology model was validated using several measures. It was found that the backbone RMSD between the final refined Model and the ET_B_ crystal structure 5 × 93 was 0.233 Å, indicating proper maintenance of the template’s structural information. Another essential requirement for any 3D model is to have good stereochemistry. For this purpose, the Procheck software was used to assess the overall quality of the protein and highlight the regions that may need further investigation. A careful assessment of the Procheck output revealed that no steric clashes or distorted geometry were present. The main chain and side chain parameters were acceptable, with no rotamer outliers. It was found that 93.9% of the residues appeared in the most favored regions of the Ramachandran plot, and 6.1% of the residues were found in the additional and generously allowed regions (Figure S13, Supplementary Materials).

A minimized structure was inserted in a pre-equilibrated DPPC lipid bilayer of 167 molecules and solvated in a cubic water box using the CHARMM-GUI online tool. The resulting system size was 80.31 X 80.31 X 127.86 Å, and the total number of atoms was 77,369, with 16,788 water molecules. The protein–membrane–water system was then equilibrated in the GROMACS software 2019 and released for 1 ns using NVT (500 ps), followed by NPT ensembles (500 ps). The resulting system was then subjected to a production MD simulation for 5 ns. The final structure after the simulation was used for molecular docking. Atrasentan and compounds **15a** and **31h** were docked into the putative binding pocket of the ET_A_ homology model using the Dock module within the MOE software. The binding site was specified using the amino acid residues Trp146, Lys166, and Arg320. Docking was performed using the default settings and the Triangular Matcher method. Docking poses were analyzed using the PyMOL Molecular Graphics Systemv2.3 (Schrödinger, LLC., New York, NY, USA).

## 4. Conclusions

In this study, we reported a straightforward method to prepare new 1,3-diaryl-5-oxo-proline derivatives. Among them, **31h** possessed the best in vitro binding profile, showing a moderate affinity for ET_A_R and selectivity over ET_B_R. The synthetic procedure adopted for the preparation of **31h**, compared with that for atrasentan, is much simpler. In particular, we showed that the 5-oxo-proline moiety may serve as a versatile and synthetically accessible scaffold for the development of ET_A_R ligands.

Finally, a comparison of the poses of the selected ligands (i.e., atrasentan, **15a**, and **31h**) inside the binding pocket of the ET_A_R homology model suggest that interaction with the key residues located in the upper and lower sub-pockets of the binding site is crucial to hitting the target. Based on these results, a further hit-to-lead optimization of compound **31h** should be performed to develop new ET_A_R ligands.

## Figures and Tables

**Table 1:**
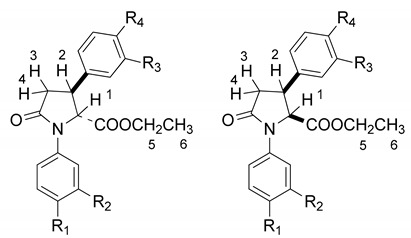
Selected ^1^H NMR (200 MHz, DMSO-*d*_6_) data of (±)-*trans* **6a**–**11a** and (±)-*cis* **6b**–1**1b** derivatives.

Compd	1 H	2 H	3 H	4 H	5 H	6 H
(±) **6a**	δ 4.84 d, *J* = 5.6 Hz	δ 3.71–3.58 m	δ 3.00 dd, ^3^*J* = 9.2 Hz, ^2^*J* = 17.0 Hz	δ 2.64 dd, ^3^*J* = 7.0 Hz, ^2^*J* = 17.0 Hz	δ 4.18–3.98 m	δ 1.04 t, *J* = 7.0 Hz
(±) **6b**	δ 5.11 d, *J* = 8.6 Hz	δ 4.17–4.07 m	δ 3.11 dd, ^3^*J* = 12.4 Hz, ^2^*J* = 16.6 Hz	δ 2.69 dd, ^3^*J* = 8.4 Hz, ^2^*J* = 16.6 Hz	δ 3.72–3.53 m	δ 0.66 t, *J* = 7.4 Hz
(±) **7a**	δ 4.79 d, *J* = 5.8 Hz	δ 3.67–3.52 m	δ 2.95 dd, ^3^*J* = 8.8 Hz, ^2^*J* = 16.8 Hz	δ 2.60 dd, ^3^*J* = 7.2 Hz, ^2^*J* = 16.8 Hz	δ 4.17–3.98 m	δ 1.05 t, *J* = 7.0 Hz
(±) **7b**	δ 5.05 d, *J* = 8.8 Hz	δ 4.16–4.01 m	δ 3.05 dd, ^3^*J* = 12.8 Hz, ^2^*J* = 16.6 Hz	δ 2.66 dd, ^3^*J* = 8.0 Hz, ^2^*J* = 16.6 Hz	δ 3.70–3.55 m	δ 0.71 t, *J* = 7.2 Hz
(±) **8a**	δ 4.82 d, *J* = 6.4 Hz	δ 3.70–3.48 m	δ 2.92 dd, ^3^*J* = 9.0 Hz, ^2^*J* = 16.8 Hz	δ 2.62 dd, ^3^*J* = 7.8 Hz, ^2^*J* = 16.8 Hz	δ 4.16–3.96 m	δ 1.03 t, *J* = 7.2 Hz
(±) **8b**	δ 5.06 d, *J* = 8.6 Hz	δ 4.15–4.01 m	δ 3.06 dd, ^3^*J* = 13.0 Hz, ^2^*J* = 16.6 Hz	δ 2.64 dd, ^3^*J* = 8.0 Hz, ^2^*J* = 16.6 Hz	δ 3.80–3.63 m	δ 0.78 t, *J* = 7.0 Hz
(±) **9a**	δ 4.81 d, *J* = 6.0 Hz	δ 3.65–3.52 m	δ 2.92 dd, ^3^*J* = 8.6 Hz, ^2^*J* = 16.6 Hz	δ 2,62 dd, ^3^*J* = 7.4 Hz, ^2^*J* = 16.6 Hz	δ 4.17–3.99 m	δ 1.03 t, *J* = 7.0 Hz
(±) **9b**	δ 5.05 d, *J* = 8.4 Hz	δ 4.16–4.04 m	δ 3.06 dd, ^3^*J* = 12.6 Hz, ^2^*J* = 16.4 Hz	δ 2.64 dd, ^3^ *J* = 8.4 Hz, ^2^ *J* = 16.4 Hz	δ 3.80–3.67 m	δ 0.78 t, *J* = 7.2 Hz
(±) **10a**	δ 4.83 d, *J* = 6.2 Hz	δ 3.68–3.48 m	δ 2.94 dd, ^3^*J* = 8.8 Hz, ^2^*J* = 16.2 Hz	δ 2.66 dd, ^3^*J* = 8.0 Hz, ^2^*J* = 16.2 Hz	δ 4.14–4.00 m	δ 1.04 t, *J* = 7.0 Hz
(±) **10b**	δ 5.07 d, *J* = 8.6 Hz	δ 4.18–4.02 m	δ 3.10 dd, ^3^*J* = 12.4 Hz, ^2^*J* = 16.4 Hz	δ 2.65 dd, ^3^*J* = 8.4 Hz, ^2^*J* = 16.4 Hz	δ 3.72–3.63 m	δ 0.73 t, *J* = 7.2 Hz
(±) **11a**	δ 4.83 d, *J* = 5.8 Hz	δ 3.68–3.52 m	δ 2.96 dd, ^3^*J* = 8.6 Hz, ^2^*J* = 16.8 Hz	δ 2.62 dd, ^3^*J* = 7.4 Hz, ^2^*J* = 16.8 Hz	δ 4.15–3.94 m	δ 1.05 t, *J* = 7.0 Hz
(±) **11b**	δ 5.08 d, *J* = 8.6 Hz	δ 4.19–4.02 m	δ 3.08 dd, ^3^*J* = 12.2 Hz, ^2^*J* = 17.0 Hz	δ 2.66 dd, ^3^*J* = 8.6 Hz, ^2^*J* = 17.0 Hz	δ 3.69–3.59 m	δ 0.73 t, *J* = 7.2 Hz

**Table 2:** Binding properties of compounds **12a**–**15a** and **31a**–**k**.

Compd	ET_Ah_ 10^−5^ M ^a^	ET_Bh_ 10^−5^ M ^a^
**12a**	NA ^b^	14
**13a**	16	NA
**14a**	11	NA
**15a**	>100	>100
**31a**	NA	NA
**31b**	NA	NA
**31c**	NA	NA
**31d**	NA	NA
**31e**	NA	NA
**31f**	NA	NA
**31g**	NA	NA
**31h**	3.3 ± 1.1 ^c^	>100
**31i**	NA	NA
**31j**	NA	NA
**31k**	NA	NA
**BQ-123**	0.0054 ± 0.0008 ^c^	NA
**BQ-788**	NA	0.0071 ± 0.0023 ^c^

^a^ Values are the percentage of inhibition of the specific binding and are the mean of three separate experiments. ^b^ NA = not active. ^c^ *K*_i_ (µM) ± SD value.

## Supplementary Materials

The following materials are available online. Synthetic procedures and experimental data of intermediate compounds **3**, **4**, (±) **6a**–**11a**, (±) **6b**–**11b**, **20**, **21**, **24**, **25**, (±) **28a**–**k**, (±) **29a**–**k**, (±) **30a**–**k**; Figures S1–S12: Representative NMR spectra for title compounds; Figure S13: Ramachandran plot analysis of the final refined model.
