# Supplementary material for: Synthesis and Molecular Modelling Studies of New 1,3-Diaryl-5-Oxo-Proline Derivatives as Endothelin Receptor Ligands
Source: Molecules. 2020 Apr 17;25(8):1851. doi: 10.3390/molecules25081851 (PMC7221592; doi:10.3390/molecules25081851)
Supplement: Supplementary file 1 [file molecules-25-01851-s001.pdf]

molecules

MDPI

Supplementary Material

Synthesis and Molecular Modelling Studies of New 1,3-Diaryl-5-Oxo-Proline Derivatives as Endothelin Receptor Ligands

Sebastiano Intagliata [1], Mohamed A. Helal [2,3], Luisa Materia [1], Valeria Pittalà [1], Loredana Salerno [1], Agostino Marrazzo [1], Alfredo Cagnotto [4], Mario Salmona [4], Maria N. Modica [1,*], and Giuseppe Romeo [1]

[1] Department of Drug Sciences, University of Catania, Viale A. Doria 6, 95125, Catania, Italy; s.intagliata@unict.it (S.I.); lmateria@yahoo.it (L.M.); vpittala@unict.it (V.P.); lsalerno@unict.it (L.S.); marrazzo@unict.it (A.M.); mmodica@unict.it (M.N.M.); gromeo@unict.it (G.R.)
[2] University of Science and Technology, Biomedical Sciences Program, Zewail City of Science and Technology, October Gardens, 6th of October, Giza, 12578, Egypt; mhelal@zewailcity.edu.eg
[3] Medicinal Chemistry Department, Faculty of Pharmacy, Suez Canal University, Ismailia 41522, Egypt.
[4] Istituto di Ricerche Farmacologiche "Mario Negri", IRCCS. Via Mario Negri, 2, 20156 Milano, Italy. alfredo.cagnotto@marionegri.it (A.C.); mario.salmona@marionegri.it (M.S.)

* Correspondence: mmodica@unict.it; Tel.: +39-095-738-6061

Table of contents

Synthetic procedures and experimental data of intermediate compounds **3**, **4**, (±) **6a–11a**, (±) **6b–11b**, **20**, **21**, **24**, **25**, (±) **28a–k**, (±) **29a–k**, (±) **30a–k**.	S2–S13
**Figures S1–S12**: Representative NMR spectra for title compounds	S14–S19
**Figure S13**: Ramachandran plot analysis of the final refined model	S20

1. [(1,3-Benzodioxol-5-yl)amino]-propanedioic acid 1,3-diethyl ester (**3**)

A mixture of 1,3-benzodioxol-5-amine (**1**) (3.00 g, 21.87 mmol) and 2-bromo-propanedioic acid 1,3-diethyl ester (2.61 g, 10.92 mmol) without solvent was prepared and stirred at room temperature under nitrogen and protected from light for 3 days. The obtained sticky product was suspended in diethyl ether (50 mL) and filtered. The organic solution was washed with HCl 1N and dried over anhydrous sodium sulfate. Successively, after the addition of animal charcoal and filtration of the suspension, the solvent was evaporated to dryness. Compound **3** (2.50 g, 77%) was obtained as a pure oil, and it was used for the next step without further purification. IR (NaCl, cm^{-1}, selected lines): 2350, 2306, 1737, 1635, 1504, 1207, 1039, 931. ^{1}H NMR ($CDCl_3$): δ 6.68-6.61 (m, 1H, Ar), 6.33-6.29 (m, 1H, Ar), 6.12-6.05 (m, 1H, Ar), 5.86 (s, 2H, OCH_2O), 4.66 (s, 1H, C*H*NH), 4.27 (q, *J* = 7.2 Hz, 2H + 2H, CH_2), 1.28 (t, *J* = 7.2 Hz, 3H + 3H, CH_3). Anal. Calcd for ($C_{14}H_{17}NO_6$): C, 56.94; H, 5.80; N, 4.74. Found: C, 56.83; H, 5.70; N, 4.85.

2. [(3,4-Dimethoxyphenyl)amino]-propanedioic acid 1,3-diethyl ester (**4**)

The title compound was prepared, following the same procedure of compound **3**, starting from 3,4-dimethoxyaniline (**2**). The obtained crude oil was purified by flash column chromatography using ethyl acetate/cyclohexane (4:6, v:v) as eluent. The solvent of homogeneous fractions was evaporated, and the residue by treatment with petroleum ether (50 mL) gave a solid which was collected and dried. Compound **4** was obtained pure (58%), mp 53-57 °C. IR (KBr, cm^{-1}, selected lines): 3369, 2982, 1722, 1516, 1186, 1024, 836, 632. ^{1}H NMR ($CDCl_3$): δ 6.79-6.68 (m, 1H, Ar), 6.39-6.31 (m, 1H, Ar), 6.22-6.12 (m, 1H, Ar), 4.71 (s, 1H, C*H*NH), 4.27 (q, *J* = 7.0 Hz, 2H + 2H, CH_2), 3.85 (s, 3H, OCH_3), 3.80 (s, 3H, OCH_3), 1.29 (t, *J* = 7.0 Hz, 3H + 3H, CH_3). Anal. Calcd for ($C_{15}H_{21}NO_6$): C, 57.87; H, 6.80; N, 4.50. Found: C, 57.90; H, 6.69; N, 4.61.

3. General procedure for the synthesis of 1,3-disubstituted-5-oxo-proline ethyl esters **6–11**

A solution of suitable diester (**3**-**5**) (3.39 mmol) in ethanol (2 ml) and a properly substituted 3-phenyl-2-propenoic acid ethyl ester (3.39 mmol) were added to a solution of EtONa, prepared by adding carefully freshly cut Na (3.39 mmol) to dry ethanol (4 mL) in a flask under reflux condenser (exothermic reaction). The mixture was kept at reflux and under stirring for 20 h under nitrogen. After being cooled, ice (15 mL) was added and the mixture was neutralized with HCl conc., and extracted with diethyl ether (3 × 15 mL). The organic layer was collected and dried over anhydrous sodium sulfate. The solvent was removed to dryness under reduced pressure to obtain desired crude products (**6–11**), which were purified by flash column chromatography using ethyl acetate/cyclohexane (4:6, v:v) as eluent. Evaporation of the solvent of homogeneous fractions gave the final compounds (±) **6a–11a** and (±) **6b–11b**. The following compounds were obtained:

3.1. 1-(4-Methoxyphenyl)-3-phenyl-5-oxo-proline ethyl esters

(±)-*trans* **6a** was obtained as a pure oil (11%). IR (NaCl, cm^{-1}, selected lines): 2979, 1705, 1512, 1249, 1191, 1029, 831, 701. ^{1}H NMR (DMSO-d_6): δ 7.44-7.29 (m, 5H + 2H, Ar), 6.99-6.90 (m, 2H, Ar), 4.84 (d, *J* = 5.6 Hz, 1H, CHCOO), 4.18-3.98 (m, 2H, C$H_2$$CH_3$), 3.73 (s, 3H, OCH_3), 3.71-3.58 (m, 1H, CHAr), 3.00 (dd, ^{3}J = 9.2 Hz; ^{2}J = 17.0 Hz, 1H, C$H_A$$H_B$CHAr), 2.64 (dd, ^{3}J = 7.0 Hz; ^{2}J = 17.0 Hz, 1H, $CH_A$$H_B$CHAr), 1.04 (t, *J* = 7.0 Hz, 3H, CH_3). Anal. Calcd for ($C_{20}H_{21}NO_4$): C, 70.78; H, 6.24; N, 4.13. Found: C, 70.93; H, 6.13; N, 4.18.

(±)-*cis* **6b** was obtained as a pure oil (1.1%). ^{1}H NMR (DMSO-d_6): δ 7.45-7.26 (m, 5H + 2H, Ar), 7.02-6.89 (m, 2H, Ar), 5.11 (d, *J* = 8.6 Hz, 1H, CHCOO), 4.17-4.07 (m, 1H, CHAr), 3.74 (s, 3H, OCH_3), 3.72-3.53 (m, 2H, C$H_2$$CH_3$), 3.11 (dd, ^{3}J = 12.4 Hz; ^{2}J = 16.6 Hz, 1H, C$H_A$$H_B$CHAr), 2.69 (dd, ^{3}J = 8.4 Hz;

^{2}J = 16.6 Hz, 1H, CH$_A$*H*$_B$CHAr), 0.66 (t, *J* = 7.4 Hz, 3H, CH$_3$). Anal. Calcd. for ($C_{20}H_{21}NO_4$): C, 70.78; H, 6.24; N, 4.13. Found: C, 70.64; H, 6.09; N, 4.20.

3.2. 1-(1,3-Benzodioxol-5-yl)-3-(4-methoxyphenyl)-5-oxo-proline ethyl esters

(±)-*trans* **7a** was obtained as a pure oil (10%), mp: 71-74 °C. IR (KBr, cm^{-1}, selected lines): 1739, 1705, 1490, 1250, 1194, 1035, 930, 835. ^{1}H NMR (DMSO-*d*$_6$): δ 7.35-7.26 (m, 2H, Ar), 7.14-7.09 (m, 1H, Ar), 6.98-6.87 (m, 3H, Ar), 6.82-6.74 (m, 1H, Ar), 6.02 (s, 2H, OCH$_2$O), 4.79 (d, *J* = 5.8 Hz, 1H, CHCOO), 4.17-3.98 (m, 2H, C*H*$_2$CH$_3$), 4.01 (s, 3H, OCH$_3$), 3.67-3.52 (m, 1H, CHAr), 2.95 (dd, ^{3}J = 8.8 Hz; ^{2}J = 16.8 Hz, 1H, C*H*$_A$H$_B$CHAr), 2.60 (dd, ^{3}J = 7.2 Hz; ^{2}J = 16.8 Hz, 1H, CH$_A$*H*$_B$CHAr), 1.05 (t, *J* = 7.0 Hz, 3H, CH$_3$). Anal. Calcd. for ($C_{21}H_{21}NO_6$): C, 65.79; H, 5.52; N, 3.65. Found: C, 65.72; H, 5.61; N, 3.73.

(±)-*cis* **7b** was obtained pure (18%). ^{1}H NMR (DMSO-*d*$_6$): δ 7.23-7.17 (m, 2H, Ar), 6.98-6.86 (m, 2H + 2H, Ar), 6.83-6.74 (m, 1H, Ar), 6.02 (s, 2H, OCH$_2$O), 5.05 (d, *J* = 8.8 Hz, 1H, CHCOO), 4.16-4.01 (m, 1H, CHAr), 3.73 (s, 3H, OCH$_3$), 3.70-3.55 (m, 2H, C*H*$_2$CH$_3$), 3.05 (dd, ^{3}J = 12.8 Hz; ^{2}J = 16.6 Hz, 1H, C*H*$_A$H$_B$CHAr), 2.66 (dd, ^{3}J = 8.0 Hz; ^{2}J = 16.6 Hz, 1H, CH$_A$*H*$_B$CHAr), 0.71 (t, *J* = 7.2 Hz, 3H, CH$_3$). Anal. Calcd. for ($C_{21}H_{21}NO_6$): C, 65.79; H, 5.52; N, 3.65. Found: C, 65.53; H, 5.66; N, 3.50.

3.3. 1,3-Di(1,3-benzodioxol-5-yl)-5-oxo-proline ethyl esters

(±)-*trans* **8a** was obtained pure (11%), mp 121-125 °C. IR (KBr, cm^{-1}, selected lines): 2977, 2915, 1705, 1485, 1252, 1036, 931, 813. ^{1}H NMR (DMSO-*d*$_6$): δ 7.13-7.03 (m, 1H + 1H, Ar), 6.94-6.73 (m, 2H + 2H, Ar), 6.01 (s, 2H + 2H, OCH$_2$O), 4.82 (d, *J* = 6.4 Hz, 1H, CHCOO), 4.16-3.96 (m, 2H, C*H*$_2$CH$_3$), 3.70-3.48 (m, 1H, CHAr), 2.92 (dd, ^{3}J = 9.0 Hz; ^{2}J = 16.8 Hz, 1H, C*H*$_A$H$_B$CHAr), 2.62 (dd, ^{3}J = 7.8 Hz; ^{2}J = 16.8 Hz, 1H, CH$_A$*H*$_B$CHAr), 1.03 (t, *J* = 7.2 Hz, 3H, CH$_3$). Anal. Calcd. for $C_{21}H_{19}NO_7$): C, 63.47; H, 4.82; N, 3.52. Found: C, 63.35; H, 4.89; N, 3.44.

(±)-*cis* **8b** was obtained pure (8%). ^{1}H NMR (DMSO-*d*$_6$): δ 7.20-7.15 (m, 1H, Ar), 6.96-6.84 (m, 3H, Ar), 6.84-6.73 (m, 2H, Ar), 6.03 (s, 2H, OCH$_2$O), 5.99 (s, 2H, OCH$_2$O), 5.06 (d, *J* = 8.6 Hz, 1H, CHCOO), 4.15-4.01 (m, 1H, CHAr), 3.80-3.63 (m, 2H, C*H*$_2$CH$_3$), 3.06 (dd, ^{3}J = 13.0 Hz; ^{2}J = 16.6 Hz, 1H, C*H*$_A$H$_B$CHAr), 2.64 (dd, ^{3}J = 8.0 Hz; ^{2}J = 16.6 Hz, 1H, CH$_A$*H*$_B$CHAr), 0.78 (t, *J* = 7.0 Hz, 3H, CH$_3$). Anal. Calcd. for ($C_{21}H_{19}NO_7$): C, 63.47; H, 4.82; N, 3.52. Found: C, 63.66; H, 4.90; N, 3.40.

3.4. 3-(1,3-Benzodioxol-5-yl)-1-(4-methoxyphenyl)-5-oxo-proline ethyl esters

(±)-*trans* **9a** was obtained pure (22%), mp 86-88 °C. IR (KBr, cm^{-1}, selected lines): 1698, 1516, 1378, 1249, 1181, 1031, 845, 634. ^{1}H NMR (DMSO-*d*$_6$): δ 7.36-7.28 (m, 2H, Ar), 7.08-7.02 (m, 1H, Ar), 6.97-6.77 (m, 2H + 2H, Ar), 6.01 (s, 2H, OCH$_2$O), 4.81 (d, *J* = 6.0 Hz, 1H, CHCOO), 4.17-3.99 (m, 2H, C*H*$_2$CH$_3$), 3.73 (s, 3H, OCH$_3$), 3.65-3.52 (m, 1H, CHAr), 2.92 (dd, ^{3}J = 8.6 Hz; ^{2}J = 16.6 Hz, 1H, C*H*$_A$H$_B$CHAr), 2.62 (dd, ^{3}J = 7.4 Hz; ^{2}J = 16.6 Hz, 1H, CH$_A$*H*$_B$CHAr), 1.03 (t, *J* = 7.0 Hz, 3H, CH$_3$). Anal. Calcd. for ($C_{21}H_{21}NO_6$): C, 65.79; H, 5.52; N, 3.65. Found: C, 65.92; H, 5.45; N, 3.74.

(±)-*cis* **9b** was obtained pure (15%). ^{1}H NMR (DMSO-*d*$_6$): δ 7.43-7.29 (m, 2H, Ar), 7.00-6.75 (m, 2H + 3H, Ar), 5.99 (s, 2H, OCH$_2$O), 5.05 (d, *J* = 8.4 Hz, 1H, CHCOO), 4.16-4.04 (m, 1H, CHAr), 3.73 (s, 3H, OCH$_3$), 3.80-3.67 (m, 2H, C*H*$_2$CH$_3$), 3.06 (dd, ^{3}J = 12.6 Hz; ^{2}J = 16.4 Hz, 1H, C*H*$_A$H$_B$CHAr), 2.64 (dd, ^{3}J = 8.4 Hz; ^{2}J = 16.4 Hz, 1H, CH$_A$*H*$_B$CHAr), 0.78 (t, *J* = 7.2 Hz, 3H, CH$_3$). Anal. Calcd. for ($C_{21}H_{21}NO_6$): C, 65.79; H, 5.52; N, 3.65. Found: C, 65.85; H, 5.71; N, 3.46.

3.5. 3-(3,4-Dimethoxyphenyl)-1-(4-methoxyphenyl)-5-oxo-proline ethyl esters

These compounds were obtained following the general procedure adopted for **6–11** using chloroform/ethyl acetate (9:1, v:v) as eluent.

(±)-*trans* **10a** was obtained as a pure oil (10%). IR (NaCl, cm^{-1}, selected lines): 2936, 1705, 1513, 1451, 1249, 1191, 1027, 831. ^{1}H NMR (DMSO-d_6): δ 7.39-7.28 (m, 2H, Ar), 7.10-7.01 (m, 1H, Ar), 7.00-6.82 (m, 2H + 2H, Ar), 4.83 (d, J = 6.2 Hz, 1H, CHCOO), 4.14-4.00 (m, 2H, CH_2CH$_3$), 3.74 (s, 3H + 3H + 3H, OCH$_3$), 3.68-3.48 (m, 1H, CHAr), 2.94 (dd, ^{3}J = 8.8 Hz; ^{2}J = 16.2 Hz, 1H, CH_AH$_B$CHAr), 2.66 (dd, ^{3}J = 8.0 Hz; ^{2}J = 16.2 Hz, 1H, CH$_A$$H_B$CHAr), 1.04 (t, J = 7.0 Hz, 3H, CH$_3$). Anal. Calcd. for ($C_{22}H_{25}NO_6$): C, 66.15; H, 6.31; N, 3.51. Found: C, 66.01; H, 6.23; N, 3.60.

(±)-*cis* **10b** was obtained as a pure oil (7%). ^{1}H NMR (DMSO-d_6): δ 7.44-7.34 (m, 2H, Ar), 7.00-6.79 (m, 2H + 3H, Ar), 5.07 (d, J = 8.6 Hz, 1H, CHCOO), 4.18-4.02 (m, 1H, CHAr), 3.75 (s, 3H, OCH$_3$), 3.74 (s, 3H, OCH$_3$), 3.73 (s, 3H, OCH$_3$), 3.72-3.63 (m, 2H, CH_2CH$_3$), 3.10 (dd, ^{3}J = 12.4 Hz; ^{2}J = 16.4 Hz, 1H, CH_AH$_B$CHAr), 2.65 (dd, ^{3}J = 8.4 Hz; ^{2}J = 16.4 Hz, 1H, CH$_A$$H_B$CHAr), 0.73 (t, J = 7.2 Hz, 3H, CH$_3$). Anal. Calcd. for ($C_{22}H_{25}NO_6$): C, 66.15; H, 6.31; N, 3.51. Found: C, 66.29; H, 6.56; N, 3.40.

3.6. 1-(3,4-Dimethoxyphenyl)-3-(4-methoxyphenyl)-5-oxo-proline ethyl esters

These compounds were obtained following the general procedure adopted for **6–11** using chloroform/ethyl acetate (9:1, v:v) as eluent.

(±)-*trans* **11a** was obtained as a pure oil (6%). ^{1}H NMR (DMSO-d_6): δ 7.50-7.26 (m, 2H + 1H, Ar), 7.17-6.82 (m, 2H + 2H, Ar), 4.83 (d, J = 5.8 Hz, 1H, CHCOO), 4.15-3.94 (m, 2H, CH_2CH$_3$), 3.75 (s, 3H, OCH$_3$), 3.73 (s, 3H, OCH$_3$), 3.72 (s, 3H, OCH$_3$), 3.68-3.52 (m, 1H, CHAr), 2.96 (dd, ^{3}J = 8.6 Hz; ^{2}J = 16.8 Hz, 1H, CH_AH$_B$CHAr), 2.62 (dd, ^{3}J = 7.4 Hz; ^{2}J = 16.8 Hz, 1H, CH$_A$$H_B$CHAr), 1.05 (t, J = 7.0 Hz, 3H, CH$_2$CH_3). Anal. Calcd. for ($C_{22}H_{25}NO_6$): C, 66.15; H, 6.31; N, 3.51. Found: C, 66.27; H, 6.40; N, 3.42.

(±)-*cis* **11b** was obtained pure (3%). ^{1}H NMR (DMSO-d_6): δ 7.32-7.21 (m, 2H + 1H, Ar), 7.00-6.80 (m, 2H + 2H, Ar), 5.08 (d, J = 8.6 Hz, 1H, CHCOO), 4.19-4.02 (m, 1H, CHAr), 3.73 (s, 3H + 3H, OCH$_3$), 3.72 (s, 3H, OCH$_3$), 3.69-3.59 (m, 2H, CH_2CH$_3$), 3.08 (dd, ^{3}J = 12.2 Hz; ^{2}J = 17.0 Hz, 1H, CH_AH$_B$CHAr), 2.66 (dd, ^{3}J = 8.6 Hz; ^{2}J = 17.0 Hz, 1H, CH$_A$$H_B$CHAr), 0.73 (t, J = 7.2 Hz, 3H, CH$_2$CH_3). Anal. Calcd. for ($C_{22}H_{25}NO_6$): C, 66.15; H, 6.31; N, 3.51. Found: C, 66.32; H, 6.51; N, 3.60.

4. 2,5-Dihydro-1-(4-methoxyphenyl)-5-oxo-3-phenyl-1H-pyrrole-2,2-dicarboxylic acid 2,2-diethyl ester (**20**)

Method A: To a solution of amide **18** (0.050 g, 0.20 mmol) in dry DMF (2 mL) was added NaH (0.009 g, 0.30 mmol, mineral oil suspension 80% m/m). The mixture was stirred at room temperature for 1 h and then diethyl 2-bromomalonate (0.048 g, 0.20 mmol) was added. The mixture was stirred at room temperature for 4 days, then was poured on ice-cold water (30 mL) and extracted with ethyl acetate (3 × 15 mL). The organic layer was collected and dried over anhydrous sodium sulfate. The solvent was removed to dryness under reduced pressure and the crude product was purified by flash column chromatography using ethyl acetate/cyclohexane (4:6, v:v) as eluent; by evaporation of the solvent of homogeneous fractions was obtained compound **20** (0.040 g, 16%) as pure oil.

Method B: To a solution of chloride derivative **19** (1.17 g, 7.11 mmol) in dry toluene (10 mL) diester **5** (2.00 g, 7.11 mmol), triethylamine (1.44 g, 14.23 mmol), and DMAP (0.35 g, 2.84 mmol) were added. The mixture was stirred for 5 days, at room temperature under nitrogen and protected from light. Then the solvent was evaporated to dryness and the obtained compound was solubilized with ethyl acetate (70 mL). The solution was washed with HCl 1N (60 mL), $NaHCO_3$ 5% (60 mL), and finally with water (2 × 50 mL). The organic layer was collected and dried over anhydrous with sodium sulfate. The solvent was removed to dryness under reduced pressure and the obtained oil was purified by flash column chromatography using ethyl acetate/cyclohexane (4:6, v:v) as eluent; by evaporation of the solvent of homogeneous fractions was obtained compound **20** (0.46 g, 49%) as pure oil. IR (NaCl, cm^{-1}, selected lines): 2982, 1708, 1512, 1296, 1246, 1049, 831, 661. ^{1}H NMR ($CDCl_3$): δ 7.58-7.50 (m, 2H, Ar), 7.46-7.36 (m, 3H, Ar), 7.33-7.23 (m, 2H, Ar), 6.96-6.87 (m, 2H, Ar), 6.68 (s, 1H, CHCO), 4.23-4.10 (m, 2H + 2H, CH$_2$), 3.81 (s, 3H, OCH$_3$), 1.07 (t, J = 7.0 Hz, 3H + 3H, CH$_3$). Anal. Calcd. for ($C_{23}H_{23}NO_6$): C, 67.47; H, 5.66; N, 3.42. Found: C, 67.23; H, 5.51; N, 3.31.

5. 1-(4-Methoxyphenyl)-5-oxo-3-phenyl-pyrrolidine-2,2-dicarboxylic acid diethyl ester (**21**)

To a solution of diester **20** (0.17 g, 0.41 mmol) in ethanol Pd/C 10% (0.020 g) was added. The mixture is placed under hydrogen at a pressure of six bars for 17 h. Then the suspension was filtered and the solvent was evaporated to dryness under reduced pressure. The crude product was purified by flash column chromatography using ethyl acetate/cyclohexane (3:7, v:v) as eluent; by evaporation of the solvent of homogeneous fractions was obtained compound **21** pure (0.060 g, 35%), mp 107-112 °C. IR (KBr, cm^{-1}, selected lines): 2982, 1724, 1512, 1355, 1247, 1030, 827, 701. ^1H NMR ($CDCl_3$): δ 7.32-7.17 (m, 5H, Ar), 7.16-7.07 (m, 2H, Ar), 6.88-6.76 (m, 2H, Ar), 4.54 (m, 1H, CHAr), 4.11-3.98 (m, 1H, $CH_AH_BCH_3$), 3.95-3.75 (m, 2H, CH_2CH_3), 3.72 (s, 3H, OCH_3), 3.48-3.36 (m, 1H, $CH_AH_BCH_3$), 2.99 (dd, ^3J = 9.8 Hz; ^2J = 17.0 Hz, 1H, CH_AH_BCHAr), 2.88 (dd, ^3J = 9.2 Hz; ^2J = 17.0 Hz, 1H, CH_AH_BCHAr), 0.89 (t, J = 7.2 Hz, 3H, CH_3), 0.71 (t, J = 7.0 Hz, 3H, CH_3). Anal. Calcd. for ($C_{23}H_{25}NO_6$): C, 67.14; H, 6.12; N, 3.40. Found: C, 67.30; H, 6.00; N, 3.51.

6. 3-(3,4-Dimethoxyphenyl)-5-oxo-pyrrolidine-2,2-dicarboxylic acid diethyl ester (**24**)

A solution of ester (**22**) (5.21 g, 22.05 mmol) in dry ethanol (12 mL) drop by drop and a solution of 2-(acetylamino)-propanedioic acid 1,3-diethyl ester (3.00 g, 13.81 mmol) in dry ethanol (6.0 mL) were added to a solution of NaH (0.10 g, 4.35 mmol) in dry ethanol (3.0 mL). The mixture was refluxed under stirring and nitrogen atmosphere for 40 h. After being cooled, the mixture was neutralized with HCl conc. and extracted with dichloromethane (3 × 25 mL). The organic layer was collected and washed with a saturated NaCl solution (50 mL). Then, the organic layer was again collected and was dried over anhydrous sodium sulfate. By evaporation of the solvent to dryness was obtained an oil, which was purified by flash column chromatography using ethyl acetate/cyclohexane (7:3, v:v) as eluent; by evaporation of the solvent of homogeneous fractions was obtained compound **24** pure (1.51 g, 30%); mp 123-126 °C. IR (KBr, cm^{-1}, selected lines): 3212, 2981, 1738, 1706, 1517, 1261, 853, 736, 653.^1H NMR($CDCl_3$): δ 6.88-6.75 (m, 3H, Ar), 6.42 (br s, 1H, NH exchanges with D_2O), 4.40-4.19 (m, 1H + 2H, CHAr + $CH_AH_BCH_3$), 3.98-3.66 (m, 2H, $CH_AH_BCH_3$), 3.86 (s, 3H, OCH_3), 3.85 (s, 3H, OCH_3), 2.96 (dd, ^3J = 8,8 Hz, ^2J = 17.4 Hz, 1H, $COCH_AH_B$), 2.62 (dd, ^3J = 5.0 Hz, ^2J = 17.4 Hz, 1H, $COCH_AH_B$), 1.29 (t, J = 7.0 Hz, 3H, CH_2CH_3), 0.90 (t, J = 7.0 Hz, 3H, CH_2CH_3). Anal. Calcd. for ($C_{18}H_{23}NO_7$): C, 59.17; H, 6.34; N, 3.83. Found: C, 59.02; H, 6.15; N, 3.66.

7. 3-(1,3-Benzodioxol-5-yl)-5-oxo-pyrrolidine-2,2-dicarboxylic acid diethyl ester (**25**)

A solution of ester (**23**) (3.24 g, 14.71 mmol) in dry ethanol (10 mL) and a solution of 2-(acetylamino)-propanedioic acid 1,3-diethyl ester (2.00 g, 9.21 mmol) in dry ethanol (8.0 mL) were added dropwise to a solution of NaH (0.063, 2.74 mmol) in dry ethanol (2.0 mL). The mixture was refluxed under stirring and nitrogen atmosphere for 18 h. After being cooled, the mixture was neutralized with HCl conc. and extracted with dichloromethane (3 × 25 mL). The organic layer was collected and washed with a saturated NaCl solution (50 mL). Then, the organic layer was collected and dried over anhydrous sodium sulfate. By evaporation of the solvent under reduced pressure to dryness was obtained an oil, which was purified by flash column chromatography using ethyl acetate/cyclohexane (5:5, v:v) as eluent; by evaporation of the solvent of homogeneous fractions was obtained compound **25** pure (0.68 g, 21%), mp 143 °C. IR (KBr, cm^{-1}, selected lines): 3184, 2997, 1736, 1486, 1225, 1085, 930, 670. ^1H NMR ($CDCl_3$): δ 6.82-6.71 (m, 3H, Ar), 5.93 (s, 2H, OCH_2O) 4.38-4.19 (m, 1H + 2H, CHAr + CH_2CH_3), 4.02-3.84 (m, 1H, $CH_AH_BCH_3$), 3.84-3.70 (m, 1H $CH_AH_BCH_3$), 2.94 (dd, ^3J = 9.0 Hz, ^2J = 17.2 Hz, 1H, $COCH_AH_B$), 2.58 (dd, ^3J = 4.8 Hz, ^2J = 17.2 Hz, 1H, $COCH_AH_B$), 1.29 (t, J = 7.2 Hz, 3H, CH_2CH_3), 0.96 (t, J = 7.4 Hz, 3H, CH_2CH_3). Anal. Calcd. for ($C_{17}H_{19}NO_7$): C, 58.45; H, 5.48; N, 4.01. Found: C, 58.63; H, 5.61; N, 4.24.

8. General procedure for the synthesis of 1,3-disubstituted-5-oxo-pyrrolidine-2,2-dicarboxylic acid diethyl esters (**28a–k**)

To a solution of the appropriate 5-oxo-pyrrolidine-2,2-dicarboxylic acid diethyl ester derivative (**24**-**27**) (1.64 mmol) in dry DMF (6.0 mL) was added NaH (1.78 mmol, mineral oil suspension 95% m/m) and the mixture was stirred at room temperature for 30 min, successively, the suitable 1-(chloromethyl)-substitutedbenzene was added (3.26 mmol). The mixture was stirred at room temperature for 1-5 days, monitoring the reaction time by TLC, and then it was poured on ice-cold water (50 mL) and extracted with ethyl acetate (3 × 25 mL). The organic layer was collected and dried over anhydrous sodium sulfate. By evaporation of the solvent to dryness was obtained the crude product, which was purified by flash column chromatography using ethyl acetate/cyclohexane (5:5, v:v), (4:6, v:v), or (3:7, v:v) as eluent; by evaporation of the solvent of homogeneous fractions was obtained desired compound **28a**-**k**. The following compounds were prepared:

8.1. 1-[(4-Methoxyphenyl)methyl)]-3-phenyl)-5-oxo-pyrrolidine-2,2-dicarboxylic acid diethyl ester (**28a**)

The title compound was obtained as a pure oil (27%). IR (NaCl, cm^{-1}, selected lines): 3044, 2972, 2902, 1733, 1512, 1246, 1175, 1033. ^1H NMR ($CDCl_3$): δ 7.35-7.18 (m, 5H, Ar), 7.17-7.08 (m, 2H, Ar), 6.86-6.77 (m, 2H, Ar), 5.13 (d, *J* = 16.0 Hz, 1H, $C\mathit{H}_AH_BN$), 4.47-4.35 (m, 1H, CHAr), 3.39 (d, *J* = 16.0 Hz, 1H, $CH_A\mathit{H}_BN$), 4.01-3.76 (m, 2H, $C\mathit{H}_2CH_3$), 3.77 (s, 3H, OCH_3), 3.57-3.40 (m, 2H, $C\mathit{H}_2CH_3$), 3.05 (dd, ^3J = 8.8 Hz; ^2J = 17.0 Hz, 1H, $C\mathit{H}_AH_BCHAr$), 2.82 (dd, ^3J = 8.0 Hz; ^2J = 17.0 Hz, 1H, $CH_A\mathit{H}_BCHAr$), 1.02 (t, *J* = 7.2 Hz, 3H, CH_3), 0.82 (t, *J* = 7.0 Hz, 3H, CH_3). Anal. Calcd. for ($C_{24}H_{27}NO_6$): C, 67.75; H, 6.40; N, 3.29. Found: C, 67.63; H, 6.23; N, 3.10.

8.2. 3-(3,4-Dimethoxyphenyl)-1-[(4-methoxyphenylmethyl)]-5-oxo-pyrrolidine-2,2-dicarboxylic acid diethyl ester (**28b**)

The title compound was obtained as a pure oil (44%). IR (NaCl, cm^{-1}, selected lines): 2940, 1731, 1611, 1461, 1030, 857, 822, 763. ^1H NMR ($CDCl_3$): δ 7.20-7.10 (m, 2H, Ar), 6.92-6.74 (m, 2H + 3H, Ar), 5.07 (d, *J* = 16.0 Hz, 1H, $NC\mathit{H}_AH_B$), 4.40-4.30 (m, 1H, CHAr), 4.30 (d, *J* = 16.0 Hz, 1H, $NCH_A\mathit{H}_B$), 4.05-3.87 (m, 2H, $C\mathit{H}_2CH_3$), 3.85 (s, 3H, OCH_3), 3.82 (s, 3H, OCH_3), 3.78 (s, 3H, OCH_3), 3.66-3.45 (m, 2H, $C\mathit{H}_2CH_3$), 3.04 (dd, ^3J = 9.0 Hz, ^2J = 17.2 Hz, 1H, $COC\mathit{H}_AH_B$), 2.77 (dd, ^3J = 7.8 Hz, ^2J = 17.2 Hz, 1H, $COCH_A\mathit{H}_B$), 1.05 (t, *J* = 7.2 Hz, 3H, $CH_2C\mathit{H}_3$), 0.89 (t, *J* = 7.2 Hz, 3H, $CH_2C\mathit{H}_3$). Anal. Calcd. for ($C_{26}H_{31}NO_8$): C, 64.32; H, 6.44; N, 2.88. Found: C, 64.14; H, 6.55; N, 3.00.

8.3. 1-[(1,3-Benzodioxol-5-yl)methyl]-3-(3,4-dimethoxyphenyl)-5-oxo-pyrrolidine-2,2-dicarboxylic acid diethyl ester (**28c**)

The title compound was obtained as a pure oil (54%). IR (NaCl, cm^{-1}, selected lines): 2980, 1729, 1599, 1444, 1034, 926, 860, 767. ^1H NMR ($CDCl_3$): δ 6.92-6.73 (m, 2H + 1H + 1H, Ar), 6.73-6.62 (m, 2H, Ar) 5.91 (s, 2H, OCH_2O), 5.00 (d, *J* = 16.0 Hz, 1H, $NC\mathit{H}_AH_B$), 4.45-4.35 (m, 1H, CHAr), 4.27 (d, *J* = 16.0 Hz, 1H, $NCH_A\mathit{H}_B$), 4.15-3.88 (m, 2H, $C\mathit{H}_2CH_3$), 3.85 (s, 3H, OCH_3), 3.83 (s, 3H, OCH_3), 3.82-3.44 (m, 2H, $C\mathit{H}_2CH_3$), 3.03 (dd, ^3J = 9.0 Hz, ^2J = 17.2 Hz, 1H, $COC\mathit{H}_AH_B$), 2.77 (dd, ^3J = 7.8 Hz, ^2J = 17.2 Hz, 1H, $COCH_A\mathit{H}_B$), 1.11 (t, *J* = 7.2 Hz, 3H, $CH_2C\mathit{H}_3$), 0.89 (t, *J* = 7.2 Hz, 3H, $CH_2C\mathit{H}_3$). Anal. Calcd. for ($C_{26}H_{29}NO_9$): C, 62.52; H, 5.85; N, 2.80. Found: C, 62.38; H, 5.57; N, 2.98.

8.4. 1-[(4-Methoxyphenyl)methyl]-3-(4-methoxyphenyl)-5-oxo-pyrrolidine-2,2-dicarboxylic acid diethyl ester (**28d**)

The title compound was obtained as a pure oil (42%). IR (NaCl, cm^{-1}, selected lines): 2934, 1731, 1612, 1461, 1247, 1181, 1032, 827. ^1H NMR ($CDCl_3$): δ 7.19-7.06 (m, 2H + 2H, Ar), 6.92-6.74 (m, 2H +

2H, Ar), 5.09 (d, *J* = 15.7 Hz, 1H, NC*H*$_A$H$_B$), 4.40-4.31 (m, 1H, CHAr), 4.27 (d, *J* = 15.7 Hz, 1H, NCH$_A$*H*$_B$), 4.03-3.80 (m, 2H, C*H*$_2$CH$_3$), 3.77 (s, 3H, OCH$_3$), 3.76 (s, 3H, OCH$_3$), 3.63-3.38 (m, 2H, C*H*$_2$CH$_3$), 3.01 (dd, ^3J = 9.0 Hz, ^2J = 17.0 Hz, 1H, COC*H*$_A$H$_B$), 2.75 (dd, ^3J = 8.0 Hz, ^2J = 17.0 Hz, 1H, COCH$_A$*H*$_B$), 1.01 (t, *J* = 7.0 Hz, 3H, CH$_2$C*H*$_3$), 0.88 (t, *J* = 7.0 Hz, 3H, CH$_2$C*H*$_3$). Anal. Calcd. for ($C_{25}H_{29}NO_7$): C, 65.92; H, 6.42; N, 3.08. Found: C, 66.12; H, 6.61; N, 3.23.

8.5. 1-[(1,3-Benzodioxol-5-yl)methyl]-3-(4-methoxyphenyl)-5-oxo-pyrrolidine-2,2-dicarboxylic acid diethyl ester (**28e**)

The title compound was obtained as a pure solid (37%); mp: 85-87 °C. IR (KBr, cm^{-1}, selected lines): 2983, 1612, 1511, 1253, 1034, 925, 840, 570. ^{1}H NMR (CDCl$_3$): δ 7.22-7.12 (m, 2H, Ar), 6.88-6.79 (m, 2H, Ar), 6.78-6.71 (m, 1H, Ar), 6.71-6.66 (m, 2H, Ar), 5.91 (s, 2H, OCH$_2$O), 5.05 (d, *J* = 16.0 Hz, 1H, NC*H*$_A$H$_B$), 4.42-4.30 (m, 1H, CHAr), 4.25 (d, *J* = 16.0 Hz, 1H, NCH$_A$*H*$_B$), 4.07-3.83 (m, 2H, C*H*$_2$CH$_3$), 3.78 (s, 3H, OCH$_3$), 3.70-3.47 (m, 2H, C*H*$_2$CH$_3$), 3.01 (dd, ^3J = 9.0 Hz, ^2J = 17.0 Hz, 1H, COC*H*$_A$H$_B$), 2.77 (dd,^3J = 8.0 Hz, ^2J = 17.0 Hz, 1H, COH$_A$*H*$_B$), 1.07 (t, *J* = 7.2 Hz, 3H, CH$_2$C*H*$_3$), 0.89 (t, *J* = 7.2 Hz, 3H, CH$_2$C*H*$_3$). Anal. Calcd. for ($C_{25}H_{27}NO_8$): C, 63.96; H, 5.80; N, 2.98. Found: C, 63.81; H, 5.69; N, 3.17.

8.6. 1-[(3,4-Dimethoxyphenyl)methyl]-3-(4-methoxyphenyl)-5-oxo-pyrrolidine-2,2-dicarboxylic acid diethyl ester (**28f**)

The title compound was obtained as a pure solid (37%); mp 94-95 °C. IR (KBr, cm^{-1}, selected lines): 2981, 1730, 1460, 1180, 1030, 836, 766, 656. ^{1}H NMR (CDCl$_3$): δ 7.23-7.12 (m, 2H, Ar), 6.87-6.67 (m, 2H + 3H, Ar), 5.10 (d, *J* = 15.8 Hz, 1H, NC*H*$_A$H$_B$), 4.42-4.29 (m, 1H, CHAr), 4.30 (d, *J* = 15.8 Hz, 1H, NCH$_A$*H*$_B$), 4.08-3.87 (m, 2H, C*H*$_2$CH$_3$), 3.86 (s, 3H, OCH$_3$), 3.81 (s, 3H, OCH$_3$), 3.74 (s, 3H, OCH$_3$), 3.70-3.40 (m, 2H, C*H*$_2$CH$_3$), 3.04 (dd, ^3J = 8.8 Hz, ^2J = 16.9 Hz, 1H, COC*H*$_A$H$_B$), 2.77 (dd, ^3J = 7.6 Hz, ^2J = 16.9 Hz, 1H, COCH$_A$*H*$_B$), 1.03 (t, *J* = 7.2 Hz, 3H, CH$_2$C*H*$_3$), 0.89 (t, *J* = 7.2 Hz, 3H, CH$_2$C*H*$_3$). Anal. Calcd. for ($C_{26}H_{31}NO_8$): C, 64.32; H, 6.44; N, 2.88. Found: C, 64.14; H, 6.24; N, 2.95.

8.7. 1-[(6-Chloro-1,3-benzodioxol-5-yl)methyl]-3-(4-methoxyphenyl)-5-oxo-pyrrolidine-2,2-dicarboxylic acid diethyl ester (**28g**)

The title compound was obtained as a pure solid (35%); mp 133-136 °C. IR (KBr, cm^{-1}, selected lines): 2988, 1730, 1610, 1481, 1030, 926, 839, 661. ^{1}H NMR (CDCl$_3$): δ 7.28-7.16 (m, 2H, Ar), 6.90-6.78 (m, 2H + 1H, Ar), 6.62-6.58 (m, 1H, Ar), 5.96 (s, 2H, OCH$_2$O, form A), 5.93 (s, 2H, OCH$_2$O, form B), 4.93 (d, *J* = 17.0 Hz, 1H, NC*H*$_A$H$_B$), 4.55-4.44 (m, 1H, CHAr), 4.43 (d, *J* = 17.0 Hz, 1H, NCH$_A$*H*$_B$), 4.13-3.98 (m, 1H, C*H*$_A$H$_B$CH$_3$), 3.98-3.83 (m, 1H, CH$_A$*H*$_B$CH$_3$), 3.79 (s, 3H, OCH$_3$), 3.73-3.50 (m, 2H, CH$_2$CH$_3$), 3.02 (dd, ^3J = 9.2 Hz, ^2J = 17.0 Hz, 1H, COC*H*$_A$H$_B$), 2.84 (dd, ^3J = 8.8 Hz, ^2J = 17.0 Hz, 1H, COCH$_A$*H*$_B$), 1.02 (t, *J* = 7.0 Hz, 3H, CH$_2$C*H*$_3$), 0.89 (t, *J* = 7.2 Hz, 3H, CH$_2$C*H*$_3$). Anal. Calcd. for ($C_{25}H_{26}ClNO_8$): C, 59.59; H, 5.20; N, 2.78. Found: C, 59.38; H, 5.05; N, 2.51.

8.8. 3-(1,3-Benzodioxol-5-yl)-1-[(4-methoxyphenyl)methyl]-5-oxo-pyrrolidine-2,2-dicarboxylic acid diethyl ester (**28h**)

The title compound was obtained as a pure solid (36%); mp 98-100 °C. IR (KBr, cm^{-1}, selected lines): 2988, 1724, 1447, 1282, 1037, 931, 817, 655. ^{1}H NMR (CDCl$_3$): δ 7.18-7.08 (m, 2H, Ar), 6.85-6.69 (m, 2H + 3H, Ar), 5.93 (s, 2H, OCH$_2$O), 5.09 (d, *J* = 15.8 Hz, 1H, NC*H*$_A$H$_B$), 4.40-4.20 (m, 1H + 1H, NCH$_A$*H*$_B$ + CHAr), 4.00-3.87 (m, 2H, C*H*$_2$CH$_3$), 3.77 (s, 3H, OCH$_3$), 3.70-3.45 (m, 2H, C*H*$_2$CH$_3$), 3.00 (dd, ^3J = 9.0 Hz, ^2J = 17.1 Hz, 1H, COC*H*$_A$H$_B$), 2.74 (dd, ^3J = 8.2 Hz, ^2J = 17.1 Hz, 1H, COH$_A$*H*$_B$), 1.03 (t, *J* = 7.2 Hz, 3H, CH$_2$C*H*$_3$), 0.94 (t, *J* = 7.2 Hz, 3H, CH$_2$C*H*$_3$). Anal. Calcd. for ($C_{25}H_{27}NO_8$): C, 63.96; H, 5.80; N, 2.98. Found: C, 63.82; H, 5.69; N, 2.76.

8.9. 1-[(1,3-Benzodioxol-5-yl)methyl]-3-(1,3-benzodioxol-5-yl)-5-oxo-pyrrolidine-2,2-dicarboxylic acid diethyl ester (**28i**)

The title compound was obtained as a pure solid (30%); mp 118-120 °C. IR (KBr, cm^{-1}, selected lines): 2986, 1732, 1493, 1245, 1038, 928, 871, 810. ^{1}H NMR ($CDCl_3$): δ 6.78-6.56 (m, 3H + 3H, Ar), 5.93 (s, 2H, OCH_2O), 5.92 (s, 2H, OCH_2O), 5.04 (d, *J* = 15.8 Hz, 1H, $NC\mathit{H}_AH_B$), 4.43-4.28 (m, 1H, CHAr), 4.23 (d, *J* = 15.8 Hz, 1H, $NCH_A\mathit{H}_B$), 4.10-3.80 (m, 2H, $C\mathit{H}_2CH_3$), 3.75-3.52 (m, 2H, $C\mathit{H}_2CH_3$), 2.99 (dd, ^3J = 9.0 Hz, ^2J = 17.0 Hz, 1H, $COC\mathit{H}_AH_B$), 2.73 (dd, ^3J = 8.2 Hz, ^2J = 17.0 Hz, 1H, $COCH_A\mathit{H}_B$), 1.08 (t, *J* = 7.0 Hz, 3H, $CH_2C\mathit{H}_3$), 0.95 (t, *J* = 7.2 Hz, 3H, $CH_2C\mathit{H}_3$). Anal. Calcd. for ($C_{25}H_{25}NO_9$): C, 62.11; H, 5.21; N, 2.90. Found: C, 62.32; H, 5.03; N, 2.72.

8.10. 3-(1,3-Benzodioxol-5-yl)-1-[(3,4-dimethoxyphenyl)methyl]-5-oxo-pyrrolidine-2,2-dicarboxylic acid diethyl ester (**28j**)

The title compound was obtained as a pure solid (41%); mp 139-141 °C. IR (KBr, cm^{-1}, selected lines): 2938, 1730, 1511, 1170, 1033, 930, 815, 768. ^{1}H NMR ($CDCl_3$): δ 6.82-6.68 (m, 3H + 3H, Ar), 5.94 (s, 2H, OCH_2O), 5.08 (d, *J* = 15.7 Hz, 1H, $NC\mathit{H}_AH_B$), 4.29 (d, *J* = 15.7 Hz, 1H, $NCH_A\mathit{H}_B$), 4.08-3.90 (m, 2H, $C\mathit{H}_2CH_3$), 3.86 (s, 3H, OCH_3), 3.85 (s, 3H, OCH_3), 3.75-3.45 (m, 2H + 1H, $C\mathit{H}_2CH_3$ + CHAr), 3.02 (dd, ^3J = 9.0 Hz, ^2J = 17.0 Hz, 1H, $COC\mathit{H}_AH_B$), 2.74 (dd, ^3J = 7.8 Hz, ^{2}J = 17.0 Hz, 1H, $COCH_A\mathit{H}_B$), 1.04 (t, *J* = 7.2 Hz, 3H, $CH_2C\mathit{H}_3$), 0.95 (t, *J* = 7.0 Hz, 3H, $CH_2C\mathit{H}_3$). Anal. Calcd. for ($C_{26}H_{29}NO_9$): C, 62.52; H, 5.85; N, 2.80. Found: C, 62.70; H, 5.97; N, 3.01.

8.11. 3-(1,3-Benzodioxol-5-yl)-1-[(6-chloro-1,3-benzodioxol-5-yl)methyl]-5-oxo-pyrrolidine-2,2-dicarboxylic acid diethyl ester (**28k**)

The title compound was obtained as a pure solid (37%); mp 143-145 °C. IR (KBr, cm^{-1}, selected lines): 2982, 1729, 1484, 1109, 1027, 918, 729, 661. ^{1}H NMR ($CDCl_3$): δ 6.82-6.73 (m, 3H + 1H, Ar), 6.60-6.57 (m, 1H, Ar), 5.95 (s, 2H + 2H, OCH_2O), 4.92 (d, *J* = 16.7 Hz, 1H, $NC\mathit{H}_AH_B$), 4.51-4.40 (m, 1H, CHAr), 4.42 (d, *J* = 16.7 Hz, 1H, $NCH_A\mathit{H}_B$), 4.10-3.90 (m, 2H, $C\mathit{H}_2CH_3$), 3.80-3.50 (m, 2H, $C\mathit{H}_2CH_3$), 3.00 (dd, ^3J = 8.8 Hz, ^2J = 17.0 Hz, 1H, $COC\mathit{H}_AH_B$), 2.81 (dd, ^3J = 9.0 Hz, ^2J = 17.0 Hz, 1H, $COCH_A\mathit{H}_B$), 1.02 (t, *J* = 7.2 Hz, 3H, $CH_2C\mathit{H}_3$), 0.95 (t, *J* = 7.2 Hz, 3H, $CH_2C\mathit{H}_3$). Anal. Calcd. for ($C_{25}H_{24}ClNO_9$): C, 57.98; H, 4.67; N, 2.70. Found: C, 58.11; H, 4.53; N, 2.93.

9. General procedure for the synthesis of 1,3-disubstituted-5-oxo-prolines (**29a–k**)

A solution of KOH (1.25 mmol) in water (2.5 mL) was added to a solution of the appropriate diester **28a**-**k** (0.62 mmol) in ethanol (5.0 mL). The mixture was refluxed for 2-11 h, monitoring the reaction time by TLC. After being cooled, the solvent was removed at reduced pressure, water was added (2 mL) and the solution was extracted with ethyl acetate (5 mL). The water layer was collected, acidified with HCl conc. and extracted with ethyl acetate (3 × 10 mL). The organic layer of this latter extraction was separated and dried over anhydrous sodium sulfate. Evaporation of the solvent to dryness gave the crude product, which was used for the next step without purification. The following compounds were obtained with this procedure.

9.1. (±)-*trans* 1-[(4-Methoxyphenyl)methyl]-3-phenyl-5-oxo-proline (**29a**)

The title compound was obtained as a pure solid (70%).mp 92-95 °C. IR (KBr, cm^{-1}, selected lines): 2933, 1639, 1457, 1247, 1178, 1033, 912, 732. ^{1}H NMR ($CDCl_3$): δ 8.41 (br s, 1H, COOH exchanges with D_2O), 7.30-6.98 (m, 5H + 2H, Ar), 6.83-6.73 (m, 2H, Ar), 5.13 (d, *J* = 14.6 Hz, 1H, $C\mathit{H}_AH_BN$), 3.95 (d, *J* = 14.6 Hz, 1H, $CH_A\mathit{H}_BN$), 3.95 (d, *J* = 3.0 Hz, 1H, CHCOO), 3.74 (s, 3H, OCH_3), 3.65-3.55 (m, 1H, CHAr), 3.07 (dd, ^3J = 9.3 Hz, ^2J = 17.4 Hz, 1H, $C\mathit{H}_AH_BCHAr$), 2.57 (dd, ^3J = 3.7 Hz, ^2J =

17.4 Hz, 1H, CH$_A$*H*$_B$CHAr). Anal. Calcd. for ($C_{19}H_{19}NO_4$): C, 70.14; H, 5.89; N, 4.31. Found: C, 70.02; H, 5.77; N, 4.24.

9.2. 3-(3,4-Dimethoxyphenyl)-1-[(4-methoxyphenyl)methyl]-5-oxo-proline (**29b**)

The title compound was obtained as a pure semisolid product (54%). IR (NaCl, cm^{-1}, selected lines): 2937, 1735, 1515, 1247, 1028, 922, 733, 663. ^{1}H NMR ($CDCl_3$): δ 10.21 (br s, 1H, COOH exchanges with D_2O), 7.30-7.10 (m, 2H, Ar), 6.92-6.45 (m, 2H + 3H, Ar), 5.15 (d, *J* = 14.8 Hz, 1H, NC*H*$_A$H$_B$), 4.02-3.85 (m, 1H + 1H, NCH$_A$*H*$_B$ + C*H*COOH), 3.82 (s, 3H, OCH_3), 3.75 (s, 3H, OCH_3), 3.72 (s, 3H, OCH_3), 3.64-3.50 (m, 1H, CHAr), 3.10 (dd, ^3J = 9.4 Hz, ^2J = 17.4 Hz, 1H, COC*H*$_A$H$_B$), 2.59 (dd, ^3J = 3.2 Hz, ^2J = 17.4 Hz, 1H, COCH$_A$*H*$_B$). Anal. Calcd. for ($C_{21}H_{23}NO_6$): C, 65.44; H, 6.02; N, 3.63. Found: C, 65.28; H, 5.87; N, 3.50.

9.3. 1-[(1,3-Benzodioxol-5-yl)methyl]-3-(3,4-dimethoxyphenyl)-5-oxo-proline (**29c**)

The title compound was obtained as a pure oil (69%); IR (NaCl, cm^{-1}, selected lines): 2950, 1739, 1512,1246, 1031, 926, 810, 734. ^{1}H NMR ($CDCl_3$): δ 6.80-6.70 (m, 3H, Ar), 6.70-6.60 (m, 2H, Ar), 6.60-6.50 (m, 1H, Ar), 5.91 (s, 2H, OCH_2O), 5.74 (br s, 1H, COOH exchanges with D_2O), 5.12 (d, *J* = 14.7 Hz, 1H, NC*H*$_A$H$_B$), 3.97 (d, *J* = 3.0 Hz, 1H, C*H*COOH), 3.94 (d, *J* = 14.7 Hz, 1H, NCH$_A$*H*$_B$), 3.84 (s, 3H, OCH_3), 3.76 (s, 3H, OCH_3), 3.60-3.50 (m, 1H, CHAr), 3.08 (dd, ^3J = 9.2 Hz, ^2J = 17.2 Hz, 1H, COC*H*$_A$H$_B$), 2.58 (dd, ^3J = 3.0 Hz, ^2J = 17.2 Hz, 1H, COCH$_A$*H*$_B$). Anal. Calcd. for ($C_{21}H_{21}NO_7$): C, 63.15; H, 5.30; N, 3.51. Found: C, 63.29; H, 5.41; N, 3.43.

9.4. 1-[(4-Methoxyphenyl)methyl]-3-(4-methoxyphenyl)-5-oxo-proline (**29d**)

The title compound was obtained as a pure oil (58%); IR (NaCl, cm^{-1}, selected lines): 2937, 1739, 1644, 1513, 1249, 1179, 1032, 830. ^{1}H NMR ($CDCl_3$): δ 7.24-7.06 (m, 2H, Ar), 7.02-6.92 (m, 2H, Ar), 6.90-6.70 (m, 4H, Ar), 5.12 (d, *J* = 14.6 Hz, 1H, NC*H*$_A$H$_B$), 3.97-3.84 (m, 1H + 1H, NCH$_A$*H*$_B$ + C*H*COOH), 3.76 (s, 3H + 3H, OCH_3), 3.66-3.51 (m, 1H, CHAr), 3.05 (dd, ^3J = 9.4 Hz, ^2J = 17.3 Hz, 1H, COC*H*$_A$H$_B$), 2.52 (dd, ^3J = 4.4 Hz, ^2J = 17.3 Hz, 1H, COCH$_A$*H*$_B$). Anal. Calcd. for ($C_{20}H_{21}NO_5$): C, 67.59; H, 5.96; N, 3.94. Found: C, 67.42; H, 6.16; N, 3.75.

9.5. 1-[(1,3-Benzodioxol-5-yl)methyl]-3-(4-methoxyphenyl)-5-oxo-proline (**29e**)

The title compound was obtained as a pure oil (63%); IR (NaCl, cm^{-1}, selected lines): 2928, 1735, 1642, 1444, 1248, 1036, 937, 828. ^{1}H NMR ($CDCl_3$): δ 10.40 (br s, 1H, COOH exchanges with D_2O), 7.08-6.98 (m, 2H, Ar), 6.84-6.71 (m, 2H, Ar), 6.71-6.62 (m, 3H, Ar), 5.88 (s, 2H, OCH_2O), 5.09 (d, *J* = 14.6 Hz, 1H, NC*H*$_A$H$_B$), 3.98-3.82 (m, 1H + 1H, NCH$_A$*H*$_B$ + C*H*COOH), 3.75 (s, 3H, OCH_3), 3.62-3.52 (m, 1H, CHAr), 3.05 (dd, ^3J = 9.6 Hz, ^2J = 17.4 Hz, 1H, COC*H*$_A$H$_B$), 2.57 (dd, ^3J = 3.8 Hz, ^2J = 17.4 Hz, 1H, COCH$_A$*H*$_B$). Anal. Calcd. for ($C_{20}H_{19}NO_6$): C, 65.03; H, 5.18; N, 3.79. Found: C, 65.29; H, 5.30; N, 3.92.

9.6. 1-[(3,4-Dimethoxyphenyl)methyl]-3-(4-methoxyphenyl)-5-oxo-proline (**29f**)

The title compound was obtained as a pure solid (60%); mp 161-164 °C. IR (KBr, cm^{-1}, selected lines): 2940, 1724, 1515, 1184, 1028, 977, 822, 522. ^{1}H NMR ($CDCl_3$): δ 7.06-6.96 (m, 2H, Ar), 6.83-6.66 (m, 2H + 3H, Ar), 5.11 (d, *J* = 14.4 Hz, 1H, NC*H*$_A$H$_B$), 4.01-3.89 (m, 1H + 1H, NCH$_A$*H*$_B$ + C*H*COOH), 3.83 (s, 3H, OCH_3), 3.76 (s, 3H + 3H, OCH_3), 3.61-3.50 (m, 1H, CHAr), 3.06 (dd, ^3J = 9.2 Hz, ^2J = 17.4 Hz, 1H, COC*H*$_A$H$_B$), 2.57 (dd, ^3J = 3.4 Hz, ^2J = 17.4 Hz, 1H, COCH$_A$*H*$_B$). Anal. Calcd. for ($C_{21}H_{23}NO_6$): C, 65.44; H, 6.02; N, 3.63. Found: C, 65.26; H, 5.87; N, 3.76.

9.7. 1-[(6-Chloro-1,3-benzodioxol-5-yl)methyl]-3-(4-methoxyphenyl)-5-oxo-proline (**29g**)

The title compound was obtained as a pure solid (86%); mp 101-103 °C. IR (KBr, cm^{-1}, selected lines): 2908, 1735, 1510, 1247, 10671, 928, 726, 671. ^{1}H NMR (CDCl$_3$): δ 7.10-7.00 (m, 2H, Ar), 6.86-6.75 (m, 2H + 2H, Ar), 5.94 (s, 2H, OCH$_2$O), 5.05 (d, J = 14.7 Hz, 1H, NCH_AH$_B$), 4.21 (d, J = 14.7 Hz, 1H, NCH$_A$$H_B$), 3.94 (d, J = 3.2 Hz, 1H, C*H*COOH), 3.78 (s, 3H, OCH$_3$), 3.60-3.48 (m, 1H, CHAr), 3.02 (dd, ^3J = 10.0 Hz, ^2J = 17.2 Hz, 1H, COCH_AH$_B$), 2.56 (dd, ^3J = 4.2 Hz, ^2J = 17.2 Hz, 1H, COCH$_A$$H_B$). Anal. Calcd. for ($C_{20}H_{18}ClNO_6$): C, 59.49; H, 4.49; N, 3.47. Found: C, 59.67; H, 4.58; N, 3.61.

9.8. 3-(1,3-Benzodioxol-5-yl)-1-[(4-methoxyphenyl)methyl]-5-oxo-proline (**29h**)

The title compound was obtained as a pure solid (72%); mp 105-107 °C. IR (KBr, cm^{-1}, selected lines): 2905, 1736, 1509, 1247, 1034, 928, 813, 726. ^{1}H NMR (CDCl$_3$): δ 10.25 (br s, 1H, COOH exchanges with D$_2$O), 7.18-7.09 (m, 2H, Ar), 6.85-6.76 (m, 2H, Ar), 6.69-6.63 (m, 1H, Ar), 6.56-6,48 (m, 2H, Ar), 5.89 (s, 2H, OCH$_2$O), 5.13 (d, J = 14.4 Hz, 1H, NCH_AH$_B$), 3.94 (d, J = 14.8 Hz, 1H, NCH$_A$$H_B$), 3.89 (d, J = 3.4 Hz, 1H, C*H*COOH), 3.78 (s, 3H, CH$_3$), 3.58-3.48 (m, 1H, CHAr), 3.05 (dd, ^3J = 9.4 Hz, ^2J = 17.4 Hz, 1H, COCH_AH$_B$), 2.53 (dd, ^3J = 3.6 Hz, ^2J = 17.4 Hz, 1H, COCH$_A$$H_B$). Anal. Calcd. for ($C_{20}H_{19}NO_6$): C, 65.03; H, 5.18; N, 3.79. Found: C, 65.28; H, 5.03; N, 3.63.

9.9. 1-[(1,3-Benzodioxol-5-yl)methyl]-3-(1,3-benzodioxol-5-yl)-5-oxo-proline (**29i**)

The title compound was obtained as a pure solid (80%); mp 73-75 °C. IR (KBr, cm^{-1}, selected lines): 2361, 1736, 1634, 1443, 1246, 1038, 930, 807. ^{1}H NMR (CDCl$_3$): δ 6.76-6.64 (m, 2H + 1H + 1H, Ar), 6.60-6.50 (m, 2H, Ar), 5.93 (s, 2H + 2H, OCH$_2$O), 5.08 (d, J = 14.8 Hz, 1H, NCH_AH$_B$), 3.90 (d, J = 14.8 Hz, 1H, NCH$_A$$H_B$), 3.91 (d, J = 3.4 Hz, 1H, C*H*COOH), 3.56-3.45 (m, 1H, CHAr), 3.02 (dd, ^3J = 8.8 Hz, ^2J = 17.1 Hz, 1H, COCH_AH$_B$), 2.53 (dd, ^3J = 4.2 Hz, ^2J = 17.1 Hz, 1H, COCH$_A$$H_B$). Anal. Calcd. for ($C_{20}H_{17}NO_7$): C, 62.66; H, 4.47; N, 3.65. Found: C, 62.80; H, 4.62; N, 3.77.

9.10. 3-(1,3-Benzodioxol-5-yl)-1-[(3,4-dimethoxyphenyl)methyl]-5-oxo-proline (**29j**)

The title compound was obtained as a pure oil (88%); IR (NaCl, cm^{-1}, selected lines): 2951, 1735, 1511, 1237, 1033, 931, 814, 732. ^{1}H NMR (CDCl$_3$): δ 6.84-6.60 (m, 3H + 1H, Ar), 6.60-6.50 (m, 2H, Ar), 5.89 (s, 2H, OCH$_2$O), 5.14 (d, J = 14.8 Hz, 1H, NCH_AH$_B$), 4.00-3.84 (m, 1H + 1H, NCH$_A$$H_B$ + C*H*COOH), 3.82 (s, 3H, OCH$_3$), 3.79 (s, 3H, OCH$_3$), 3.56-3.46 (m, 1H, CHAr), 3.05 (dd, ^3J = 9.2 Hz, ^2J = 17.7 Hz, 1H, COCH_AH$_B$), 2.53 (dd, ^3J = 3.0 Hz, ^2J = 17.7 Hz, 1H, COCH$_A$$H_B$). Anal. Calcd. for ($C_{21}H_{21}NO_7$): C, 63.15; H, 5.30; N, 3.51. Found: C, 63.00; H, 5.09; N, 3.41.

9.11. 1-[(6-Chloro-1,3-benzodioxol-5-yl)methyl]-3-(1,3-benzodioxol-5-yl)-5-oxo-proline (**29k**)

The title compound was obtained as a pure solid (72%); mp 88-90 °C. IR (KBr, cm^{-1}, selected lines): 2904, 1736, 1645, 1481, 1243, 1037, 929, 670. ^{1}H NMR (CDCl$_3$): δ 6.82-6.78 (m, 2H, Ar), 6.74-6.68 (m, 1H, Ar), 6.62-6.55 (m, 2H, Ar), 5.96 (s, 2H, OCH$_2$O), 5.93 (s, 2H, OCH$_2$O), 5.06 (d, J = 15.0 Hz, 1H, NCH_AH$_B$), 4.21 (d, J = 15.0 Hz, 1H, NCH$_A$$H_B$), 3.94 (d, J = 3.4 Hz, 1H, C*H*COOH), 3.54-3.47 (m, 1H, CHAr), 3.00 (dd, ^3J = 9.8 Hz, ^2J = 17.5 Hz, 1H, COCH_AH$_B$), 2.52 (dd, ^3J = 4.4 Hz, ^2J = 17.5 Hz, 1H, COCH$_A$$H_B$). Anal. Calcd. for ($C_{20}H_{16}ClNO_7$): C, 57.50; H, 3.86; N, 3.35. Found: C, 57.38; H, 3.99; N, 3.48.

10. General procedure for the synthesis of (±)-*trans* 1,3-disubstituted-5-oxo-proline methyl esters **30a–k**

Thionyl chloride (0.60 mmol) was added to a solution of the appropriate acid (**29a–k**) in methanol (5.0 mL). The mixture was refluxed for 2-4 h. After being cooled, the solvent was removed and the residue extracted with ethyl acetate (3 × 20 mL). The organic layer was washed with a 5%

water solution of Na_2CO_3 (30 mL) and a saturated NaCl solution (30 mL). The organic layer was collected and dried over anhydrous sodium sulfate. By evaporation of the solvent to dryness was obtained the crude product, which was used for the next step without purification. The following compounds were obtained with this procedure.

10.1. (±)-*trans* 1-[(Methoxyphenyl)methyl]-3-phenyl-5-oxo-proline methyl ester (**30a**)

The title compound was obtained as a pure oil (81%). IR (NaCl, cm^{-1}, selected lines): 2951, 1743, 1611, 1419, 1246, 1030, 821, 763. ^{1}H NMR ($CDCl_3$): δ 7.32-7.20 (m, 3H, Ar), 7.17-7.02 (m, 2H + 2H, Ar), 6.88-6.74 (m, 2H, Ar), 5.01 (d, *J* = 14.3 Hz, 1H, NC*H*$_A$H$_B$), 4.00 (d, *J* = 14.3 Hz, 1H, NCH$_A$*H*$_B$), 3.93 (d, *J* = 3.4 Hz, 1H, C*H*$COOCH_3$), 3.77 (s, 3H, OCH_3), 3.70 (s, 3H, $COOCH_3$), 3.55-3.45 (m, 1H, CHAr), 3.03 (dd, ^3J = 9.3 Hz, ^2J = 17.2 Hz, 1H, COC*H*$_A$H$_B$), 2.55 (dd, ^3J = 4.0 Hz, ^2J = 17.2 Hz, 1H, COCH$_A$*H*$_B$). Anal. Calcd. for ($C_{20}H_{21}NO_4$): C, 70.78; H, 6.24; N, 4.13. Found: C, 70.63; H, 6.01; N, 3.97.

10.2. (±)-*trans* 3-(3,4-Dimethoxyphenyl)-1-[(4-methoxyphenyl)methyl]-5-oxo-proline methyl ester (**30b**)

The title compound was obtained as a pure oil (80%); IR (NaCl, cm^{-1}, selected lines): 2945, 1742, 1695, 1513, 1454, 1246, 1027, 873. ^{1}H NMR ($CDCl_3$): δ 7.20-7.08 (m, 2H, Ar), 6.86-6.46 (m, 2H + 3H, Ar), 5.03 (d, *J* = 14.6 Hz, 1H, NC*H*$_A$H$_B$), 3.95 (d, *J* = 14.6 Hz, 1H, NCH$_A$*H*$_B$), 3.96 (d, *J* = 3.0 Hz, 1H, C*H*$COOCH_3$), 3.83 (s, 3H, OCH_3), 3.77 (s, 3H, OCH_3), 3.74 (s, 3H, OCH_3), 3.73 (s, 3H, $COOCH_3$), 3.50-3.40 (m, 1H, CHAr), 3.04 (dd, ^3J = 9.4 Hz, ^2J = 17.4 Hz, 1H, COC*H*$_A$H$_B$), 2.53 (dd, ^3J = 3.8 Hz, ^2J = 17.4 Hz, 1H, COCH$_A$*H*$_B$). Anal. Calcd. for ($C_{22}H_{25}NO_6$): C, 66.15; H, 6.31; N, 3.51. Found: C, 66.33; H, 6.13; N, 3.39.

10.3. (±)-*trans* 1-[(1,3-Benzodioxol-5-yl)methyl]-3-(3,4-dimethoxyphenyl)-5-oxo-proline methyl ester (**30c**)

The title compound was obtained as a pure solid oil (74%); IR (NaCl, cm^{-1}, selected lines): 2950, 1695, 1443, 1243, 1031, 926, 810, 769. ^{1}H NMR ($CDCl_3$): δ 6.81-6.60 (m, 3H + 2H, Ar), 6.55-6,48 (m, 1H, Ar), 5.92 (s, 2H, OCH_2O), 5.03 (d, *J* = 14.6 Hz, 1H, NC*H*$_A$H$_B$), 3.95 (d, *J* = 3.0 Hz, 1H, C*H*$COOCH_3$), 3.87 (d, *J* = 14.6 Hz, 1H, NCH$_A$*H*$_B$), 3.85 (s, 3H, OCH_3), 3.77 (s, 3H, OCH_3), 3.75 (s, 3H, $COOCH_3$), 3.52-3,40 (m, 1H, CHAr), 3.03 (dd, ^3J = 9.6 Hz, ^2J = 17.2 Hz, 1H, COC*H*$_A$H$_B$), 2.53 (dd, ^3J = 3.8 Hz, ^2J = 17.2 Hz, 1H, COCH$_A$*H*$_B$). Anal. Calcd. for ($C_{22}H_{23}NO_7$): C, 63.91; H, 5.61; N, 3.39. Found: C, 64.19; H, 5.82; N, 3.23.

10.4. (±)-*trans* 1-[(4-Methoxyphenyl)methyl]-3-(4-methoxyphenyl)-5-oxo-proline methyl ester (**30d**)

The title compound was obtained as a pure solid oil (70%); IR (NaCl, cm^{-1}, selected lines): 2951, 1742, 1513, 1443, 1249, 1178, 1032, 830. ^{1}H NMR ($CDCl_3$): δ 7.16-7.07 (m, 2H, Ar), 7.02-6.95 (m, 2H, Ar), 6.86-6,73 (m, 4H, Ar), 4.99 (d, *J* = 14.6 Hz, 1H, NC*H*$_A$H$_B$), 3.98 (d, *J* = 14.6 Hz, 1H, NCH$_A$*H*$_B$), 3.89 (d, *J* = 3.6 Hz, 1H, C*H*$COOCH_3$), 3.78 (s, 3H, OCH_3), 3.77 (s, 3H, OCH_3), 3.69 (s, 3H, $COOCH_3$), 3.52-3.40 (m, 1H, CHAr), 3.01 (dd, ^3J = 9.4 Hz, ^2J = 17.0 Hz, 1H, COC*H*$_A$H$_B$), 2.51 (dd, ^3J = 4.4 Hz, ^2J = 17.0 Hz, 1H, COCH$_A$*H*$_B$). Anal. Calcd. for ($C_{21}H_{23}NO_5$): C, 68.28; H, 6.28; N, 3.79. Found: C, 68.11; H, 6.42; N, 3.95.

10.5. (±)-*trans* 1-[(1,3-Benzodioxol-5-yl)methyl]-3-(4-methoxyphenyl)-5-oxo-proline methyl ester (**30e**)

The title compound was obtained as a pure oil, which was purified by flash column chromatography using ethyl acetate/cyclohexane (5:5, v:v) as eluent and by evaporation of the solvent of homogeneous fractions was obtained a pure oil (71%). IR (NaCl, cm^{-1}, selected lines): 1740,

1696, 1608, 1442, 1249, 1035, 926, 829. ^{1}H NMR ($CDCl_3$): δ 7.06-6.98 (m, 2H, Ar), 6.85-6.78 (m, 2H, Ar), 6.72-6.64 (m, 3H, Ar), 5.93 (s, 2H, OCH_2O), 4.99 (d, J = 14.2 Hz, 1H, $NC\mathit{H}_AH_B$), 3.92 (d, J = 14.2 Hz, 1H, $NCH_A\mathit{H}_B$), 3.85 (d, J = 3.6 Hz, 1H, C*H*$COOCH_3$), 3.78 (s, 3H, OCH_3), 3.72 (s, 3H, $COOCH_3$), 3.53-3.42 (m, 1H, CHAr), 3.00 (dd, ^3J = 9.4 Hz, ^2J = 17.0 Hz, 1H, $COC\mathit{H}_AH_B$), 2.52 (dd, ^3J = 4.4 Hz, ^2J = 17.0 Hz, 1H, $COCH_A\mathit{H}_B$). Anal. Calcd. for ($C_{21}H_{21}NO_6$): C, 65.79; H, 5.52; N, 3.65. Found: C, 65.93; H, 5.41; N, 3.49.

10.6. (±)-*trans* 1-[(3,4-Dimethoxyphenyl)methyl]-3-(4-methoxyphenyl)-5-oxo-proline methyl ester (**30f**)

The title compound was obtained as a pure oil (84%). IR (NaCl, cm^{-1}, selected lines): 2943, 1742, 1694, 1514, 1257, 1030, 874, 832. ^{1}H NMR ($CDCl_3$): δ 7.07-6.96 (m, 2H, Ar), 6.84-6.66 (m, 2H + 3H, Ar), 5.03 (d, J = 14.6 Hz, 1H, $NC\mathit{H}_AH_B$), 3.96 (d, J = 14.6 Hz, 1H, $NCH_A\mathit{H}_B$), 3.90 (d, J = 3.0 Hz, 1H, C*H*$COOCH_3$), 3.84 (s, 3H, OCH_3), 3.78 (s, 3H, OCH_3), 3.76 (s, 3H, OCH_3), 3.72 (s, 3H, $COOCH_3$), 3.55-3.42 (m, 1H, CHAr), 3.02 (dd, ^3J = 9.4 Hz, ^2J = 17.1 Hz, 1H, $COC\mathit{H}_AH_B$), 2.53 (dd, ^3J = 3.8 Hz, ^2J = 17.1 Hz, 1H, $COCH_A\mathit{H}_B$). Anal. Calcd. for ($C_{22}H_{25}NO_6$): C, 66.15; H, 6.31; N, 3.51. Found: C, 66.02; H, 6.19; N, 3.40.

10.7. (±)-*trans* 1-[(6-Chloro-1,3-benzodioxol-5-yl)methyl]-3-(4-methoxyphenyl)-5-oxo-proline methyl ester (**30g**)

The title compound was obtained as a pure oil (86%). IR (NaCl, cm^{-1}, selected lines): 2950, 1742, 1480, 1247, 1034, 929, 874, 834. ^{1}H NMR ($CDCl_3$): δ 7.10-6.98 (m, 2H, Ar), 6.88-6.72 (m, 2H + 2H, Ar), 5.95 (s, 2H, OCH_2O), 4.99 (d, J = 14.9 Hz, 1H, $NC\mathit{H}_AH_B$), 4.20 (d, J = 14.9 Hz, 1H, $NCH_A\mathit{H}_B$), 3.91 (d, J = 3.6 Hz, 1H, C*H*$COOCH_3$), 3.78 (s, 3H, OCH_3), 3.74 (s, 3H, $COOCH_3$), 3.52-3.39 (m, 1H, CHAr), 2.98 (dd, ^3J = 9.4 Hz, ^2J = 17.2 Hz, 1H, $COC\mathit{H}_AH_B$), 2.53 (dd, ^3J = 4.4 Hz, ^2J = 17.2 Hz, 1H, $COCH_A\mathit{H}_B$). Anal. Calcd. for ($C_{21}H_{20}ClNO_6$): C, 60.36; H, 4.82; N, 3.35. Found: C, 60.50; H, 4.71; N, 3.46.

10.8. (±)-*trans* 3-(1,3-Benzodioxol-5-yl)-1-[(4-methoxyphenyl)methyl]-5-oxo-proline methyl ester (**30h**)

The title compound was obtained as a pure oil (73%). IR (NaCl, cm^{-1}, selected lines): 2943, 1742, 1695, 1508, 1246, 1035, 931, 815. ^{1}H NMR ($CDCl_3$): δ 7.19-7.09 (m, 2H, Ar), 6.86-6.78 (m, 2H, Ar), 6.70-6.62 (m, 1H, Ar), 6.56-6.47 (m, 2H, Ar), 5.90 (s, 2H, OCH_2O), 4.99 (d, J = 14.6 Hz, 1H, $NC\mathit{H}_AH_B$), 3.98 (d, J = 14.6 Hz, 1H, $NCH_A\mathit{H}_B$), 3.87 (d, J = 3.4 Hz, 1H, C*H*$COOCH_3$), 3.77 (s, 3H, OCH_3), 3.69 (s, 3H, $COOCH_3$), 3.48-3.37 (m, 1H, CHAr), 2.99 (dd, ^3J = 9.4 Hz, ^2J = 17.0 Hz, 1H, $COC\mathit{H}_AH_B$), 2.48 (dd, ^3J = 4.4 Hz, ^2J = 17.0 Hz, 1H, $COCH_A\mathit{H}_B$). Anal. Calcd. for ($C_{21}H_{21}NO_6$): C, 65.79; H, 5.52; N, 3.65. Found: C, 65.94; H, 5.41; N, 3.76.

10.9. (±)-*trans* 1-[(1,3-Benzodioxol-5-yl)methyl]-3-(1,3-benzodioxol-5-yl)-5-oxo-proline methyl ester (**30i**)

The title compound was obtained as a pure oil (87%). IR (NaCl, cm^{-1}, selected lines): 2911, 1742, 1695, 1495, 1246, 1038, 929, 812. ^{1}H NMR ($CDCl_3$): δ 6.75-6.60 (m, 2H + 1H + 1H, Ar), 6.60-6.49 (m, 2H, Ar), 5.93 (s, 2H + 2H, OCH_2O), 4.98 (d, J = 14.6 Hz, 1H, $NC\mathit{H}_AH_B$), 3.90 (d, J = 14.6 Hz, 1H, $NCH_A\mathit{H}_B$), 3.88 (d, J = 3.8 Hz, 1H, C*H*$COOCH_3$), 3.72 (s, 3H, $COOCH_3$), 3.52-3.38 (m, 1H, CHAr), 2.98 (dd, ^3J = 9.4 Hz, ^2J = 17.2 Hz, 1H, $COC\mathit{H}_AH_B$), 2.49 (dd, ^3J = 4.4 Hz, ^2J = 17.2 Hz, 1H, $COCH_A\mathit{H}_B$). Anal. Calcd. for ($C_{21}H_{19}NO_7$): C, 63.47; H, 4.82; N, 3.52. Found: C, 63.28; H, 4.72; N, 3.64.

10.10. (±)-*trans* 3-(1,3-Benzodioxol-5-yl)-1-[(3,4-dimethoxyphenyl)methyl]-5-oxo-proline methyl ester (**30j**)

The title compound was obtained as a pure oil (77%). IR (NaCl, cm^{-1}, selected lines): 2948, 1741, 1695, 1445, 1238, 1032, 930, 812. ^{1}H NMR ($CDCl_3$): δ 6.81-6.62 (m, 3H + 1H, Ar), 6.62-6.50 (m, 2H, Ar), 5.92 (s, 2H, OCH_2O), 5.05 (d, *J* = 14.7 Hz, 1H, NC*H*$_A$H$_B$), 3.93 (d, *J* = 14.7 Hz, 1H, NCH$_A$*H*$_B$), 3.88 (d, *J* = 4.4 Hz, 1H, C*H*COOCH$_3$), 3.85 (s, 3H, OCH_3), 3.82 (s, 3H, OCH_3), 3.73 (s, 3H, $COOCH_3$), 3.49-3.37 (m, 1H, CHAr), 3.01 (dd, ^3J = 9.0 Hz, ^2J = 17.2 Hz, 1H, COC*H*$_A$H$_B$), 2.50 (dd, ^3J = 3.6 Hz, ^2J = 17.2 Hz, 1H, COCH$_A$*H*$_B$). Anal. Calcd. for ($C_{22}H_{23}NO_7$): C, 63.91; H, 5.61; N, 3.39. Found: C, 63.76; H, 5.49; N, 3.50.

10.11. (±)-*trans* 3-(1,3-Benzodioxol-5-yl)-1-[(6-chloro-1,3-benzodioxol-5-yl)methyl]-5-oxo-proline methyl ester (**30k**)

The title compound was obtained as a pure oil (81%). IR (NaCl, cm^{-1}, selected lines): 2918, 1741, 1697, 1441, 1241, 1037, 928, 875. ^{1}H NMR ($CDCl_3$): δ 6.82-6.75 (m, 2H, Ar), 6.75-6.66 (m, 1H, Ar), 6.62-6.52 (m, 2H, Ar), 5.97 (s, 2H, OCH_2O, form A), 5.96 (s, 2H, OCH_2O, form B), 5.93 (s, 2H, OCH_2O), 4.99 (d, *J* = 14.8 Hz, 1H, NC*H*$_A$H$_B$), 4.20 (d, *J* = 14.8 Hz, 1H, NCH$_A$*H*$_B$), 3.90 (d, *J* = 3.4 Hz, 1H, C*H*COOCH$_3$), 3.74 (s, 3H, $COOCH_3$), 3.48-3.34 (m, 1H, CHAr), 2.96 (dd, ^3J = 9.4 Hz, ^2J = 17.2 Hz, 1H, COC*H*$_A$H$_B$), 2.49 (dd, ^3J = 4.4 Hz, ^2J = 17.2 Hz, 1H, COCH$_A$*H*$_B$). Anal. Calcd. for ($C_{21}H_{18}ClNO_7$): C, 58.41; H, 4.20; N, 3.24. Found: C, 58.56; H, 4.13; N, 3.09.

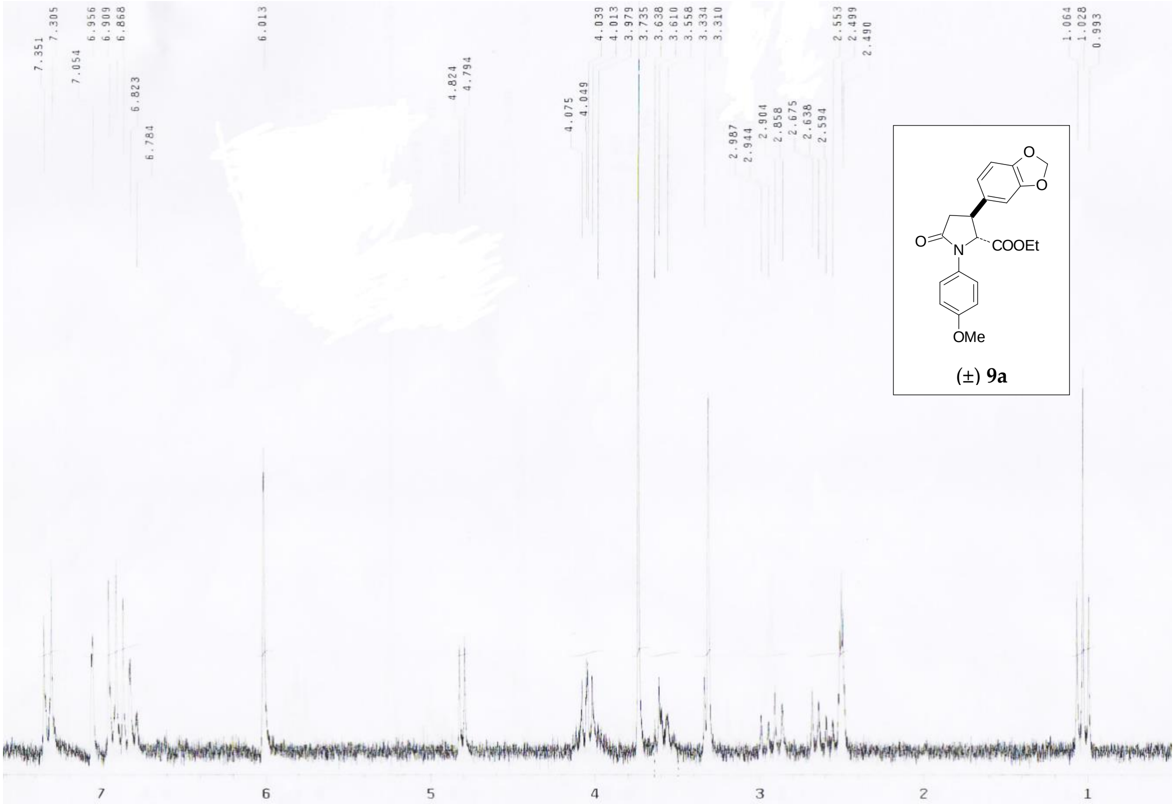

Figure S1. ^{1}H NMR spectrum of compound (±) **9a**

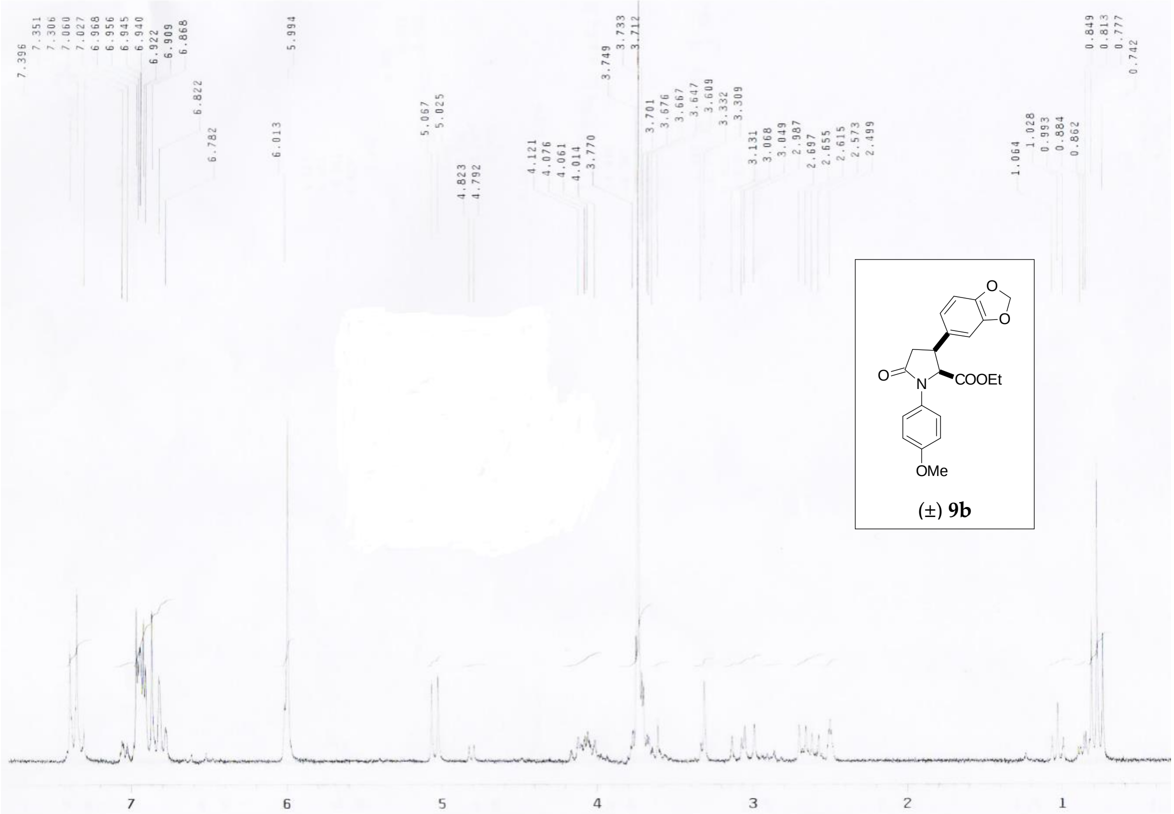

Figure S2. ^{1}H NMR spectrum of compound (±) **9b**

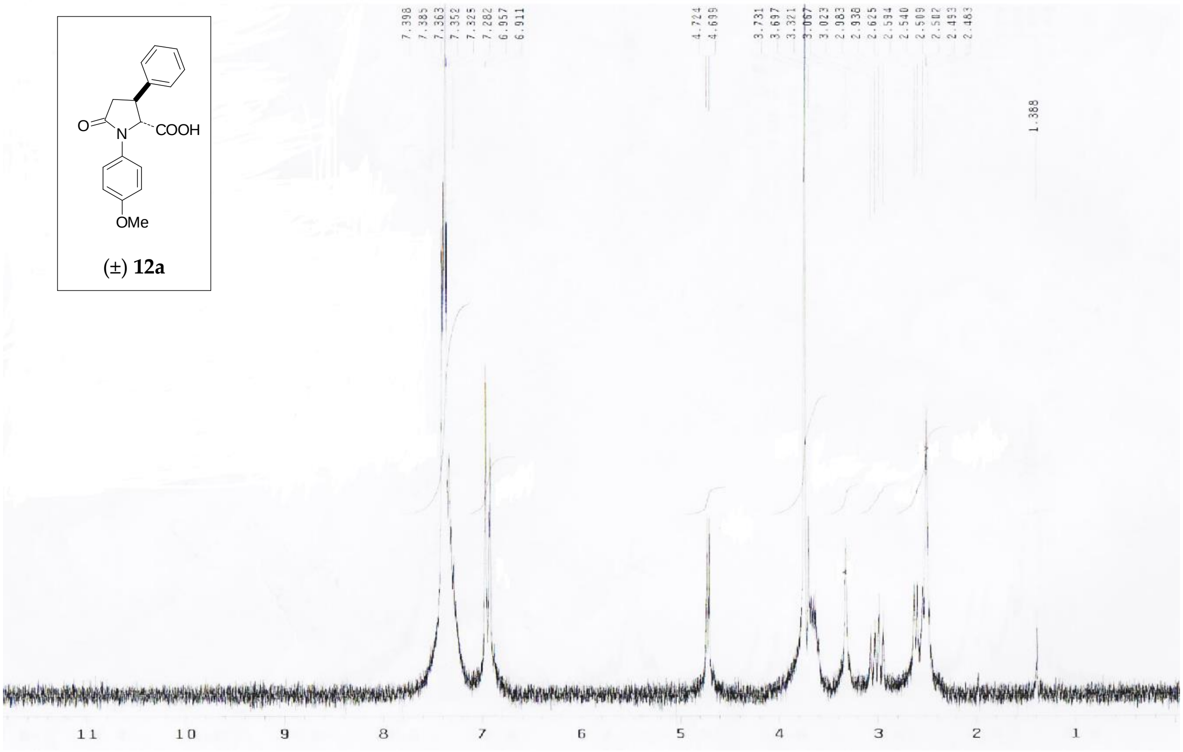

Figure S3. ^{1}H NMR spectrum of compound (±) **12a**

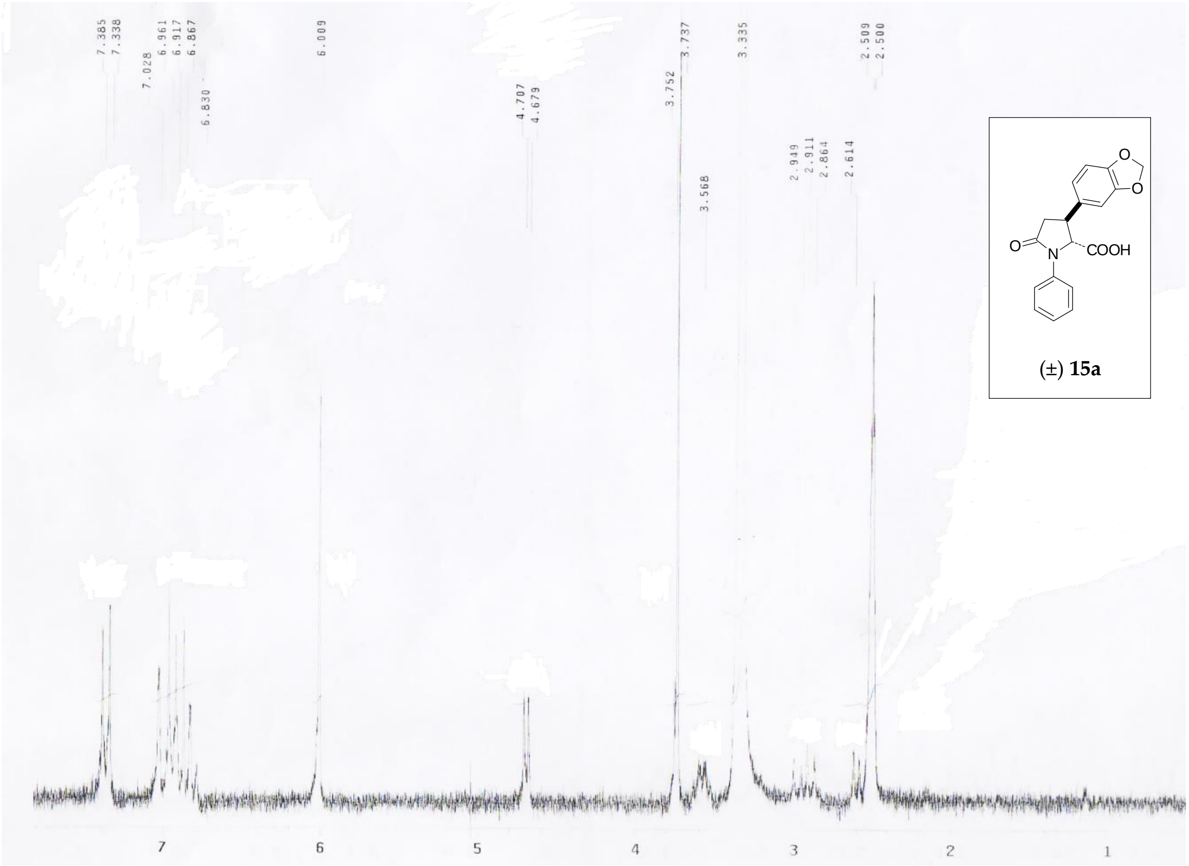

Figure S4. ^{1}H NMR spectrum of compound (±) **15a**

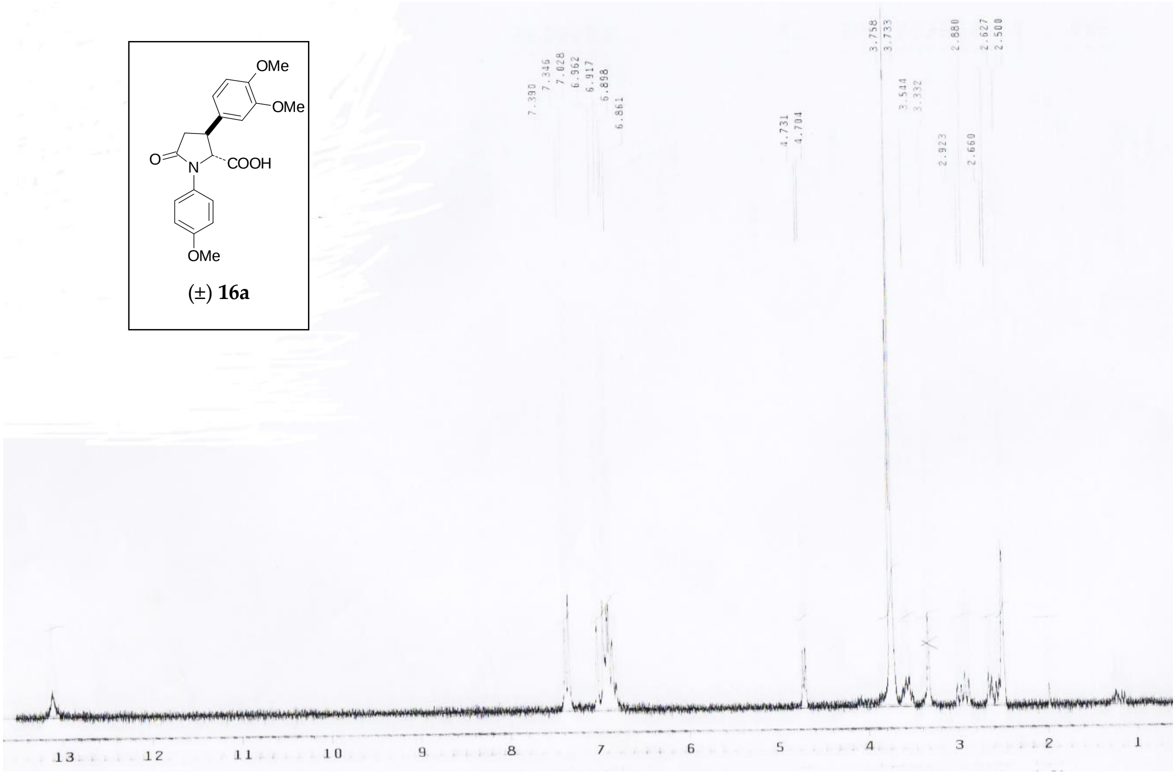

Figure S5. ^{1}H NMR spectrum of compound (±) **16a**

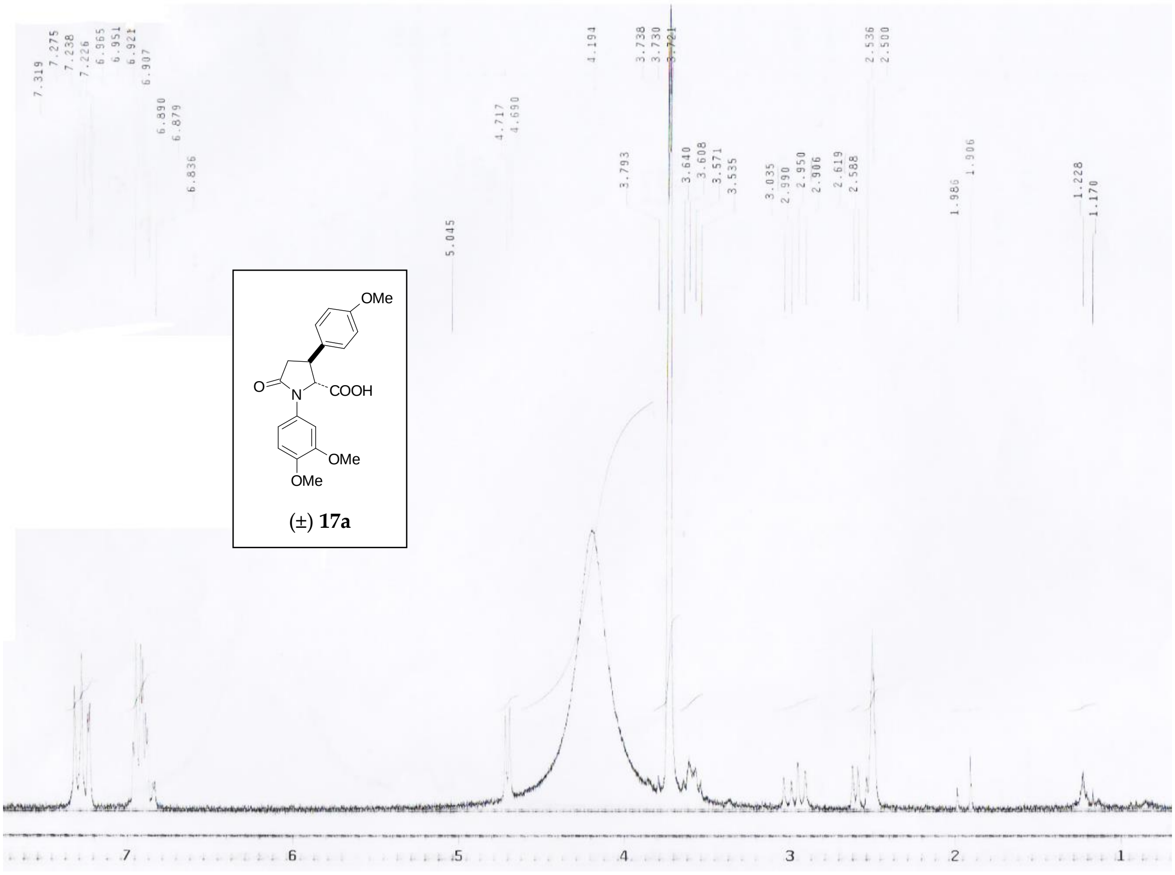

Figure S6. ^{1}H NMR spectrum of compound (±) **17a**

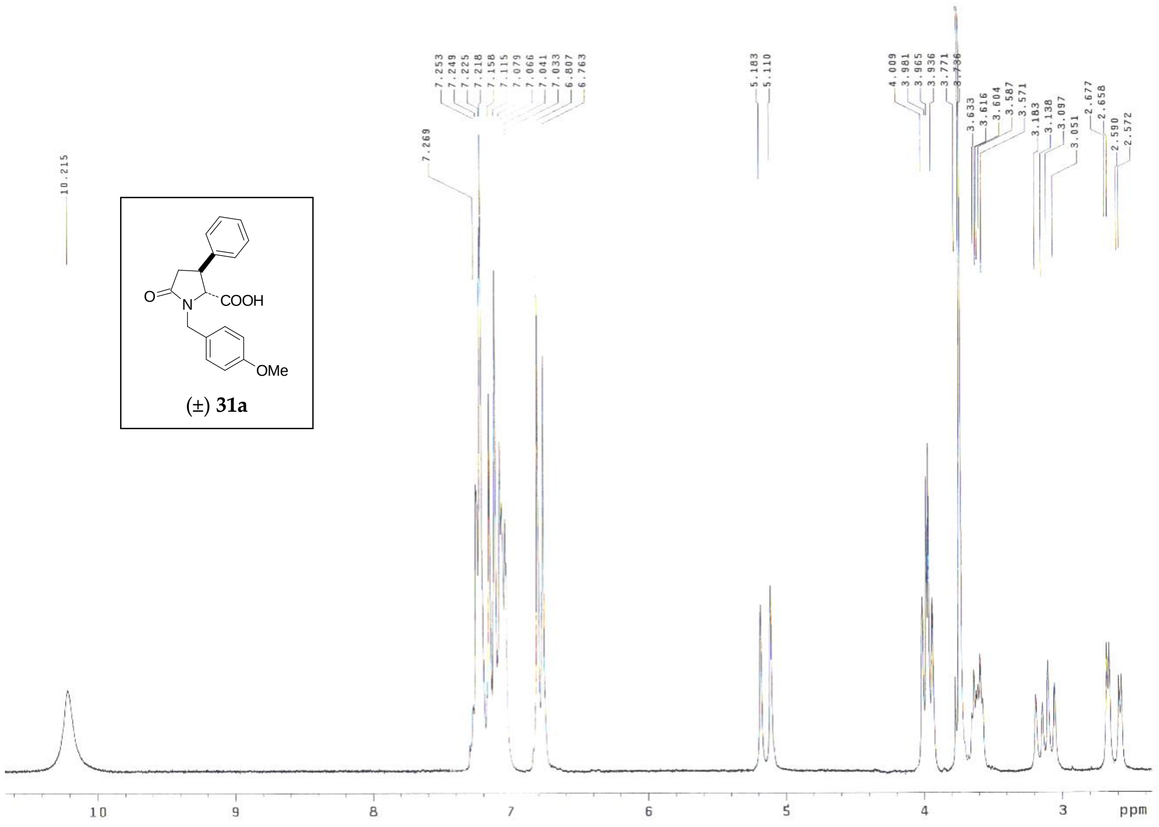

Figure S7. ^{1}H NMR spectrum of compound (±) **31a**

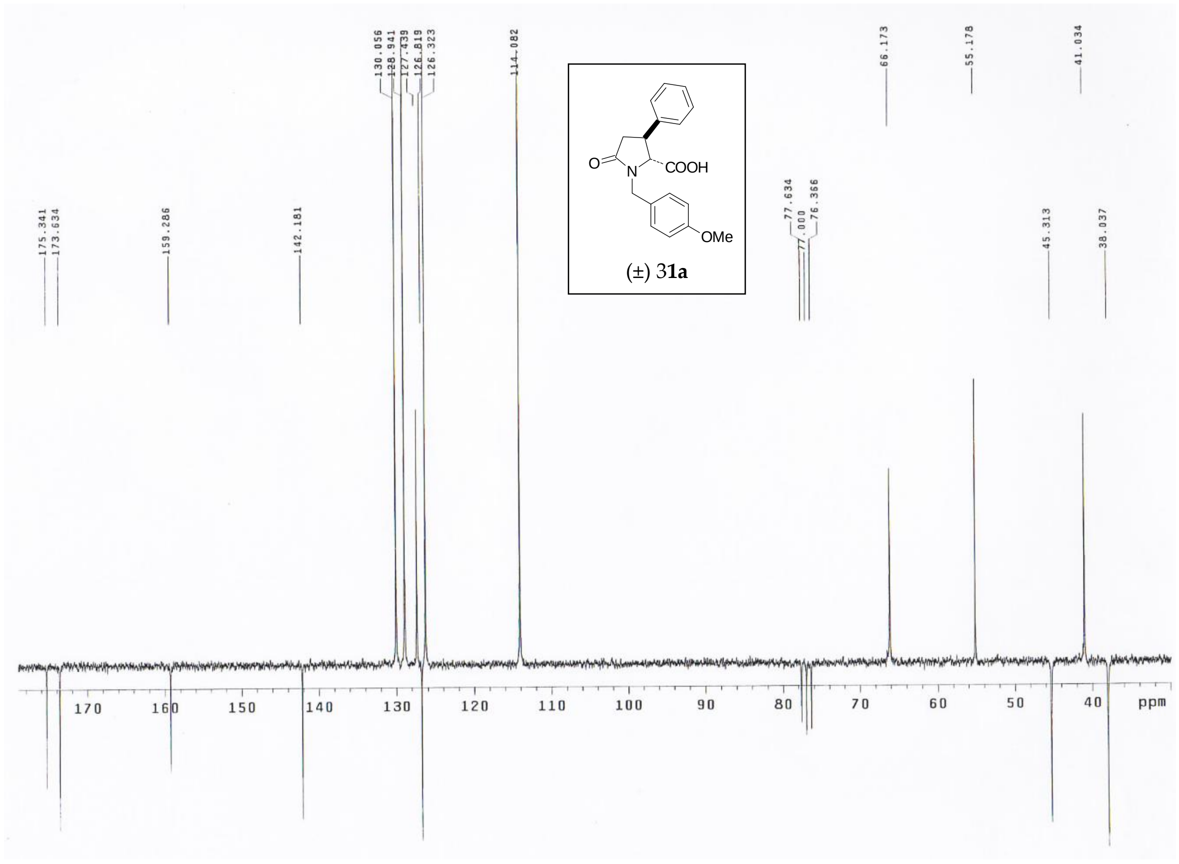

Figure S8. ^{13}C Attached Proton Test spectrum of compound (±) **31a**

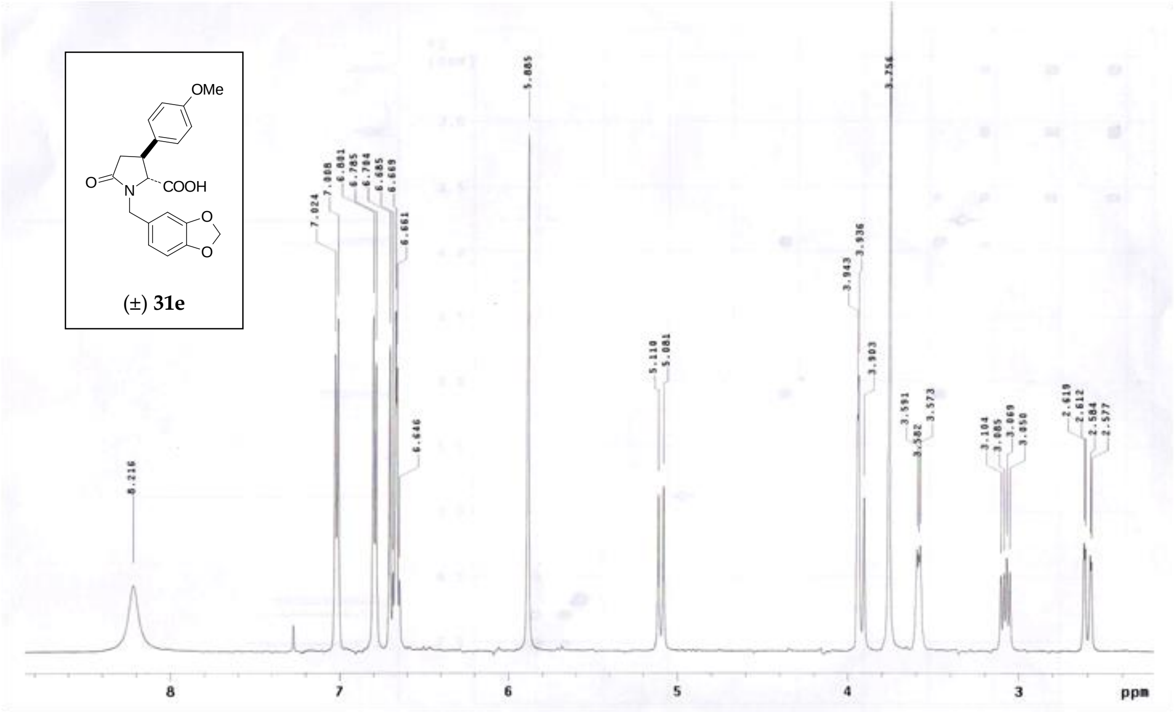

Figure S9. ^{1}H NMR spectrum of compound (±) **31e**

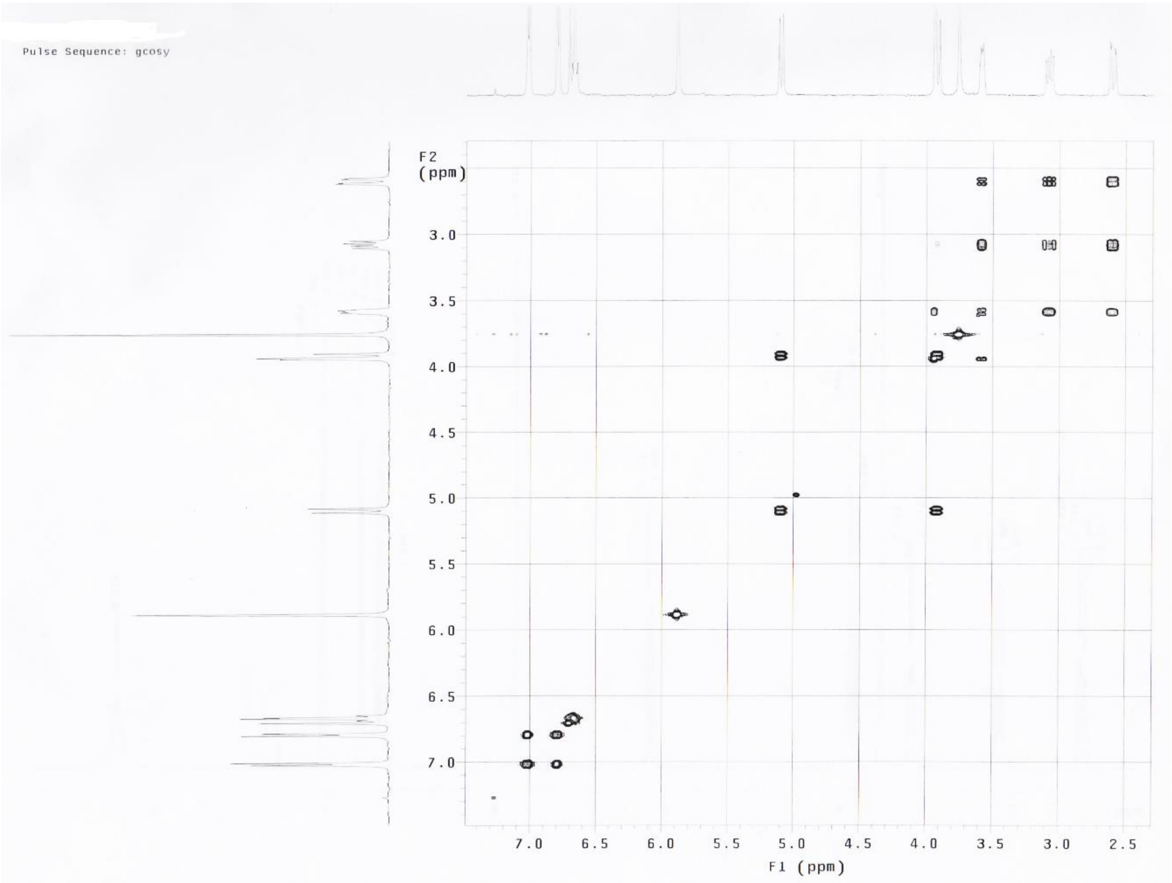

Figure S10. ^{1}H-^{1}HgCOSY spectrum of compound (±) **31e**

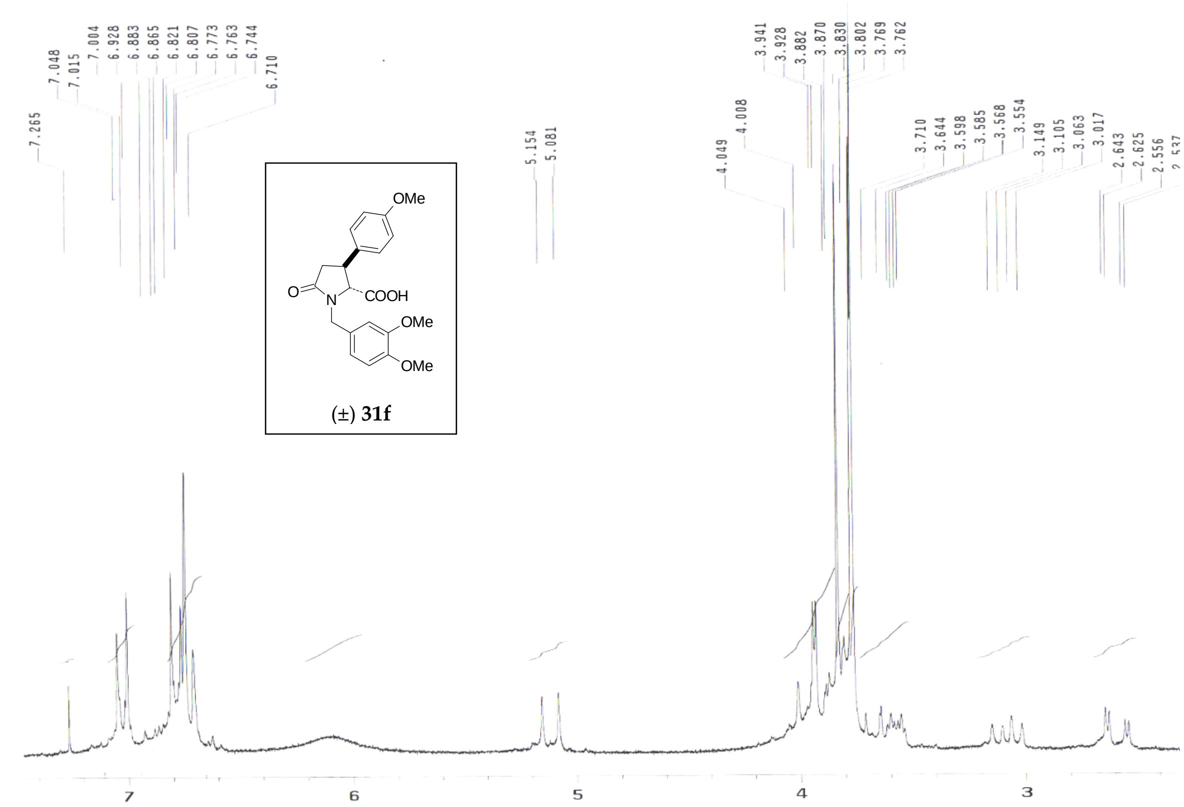

Figure S11. ^{1}H NMR spectrum of compound (±) **31f**

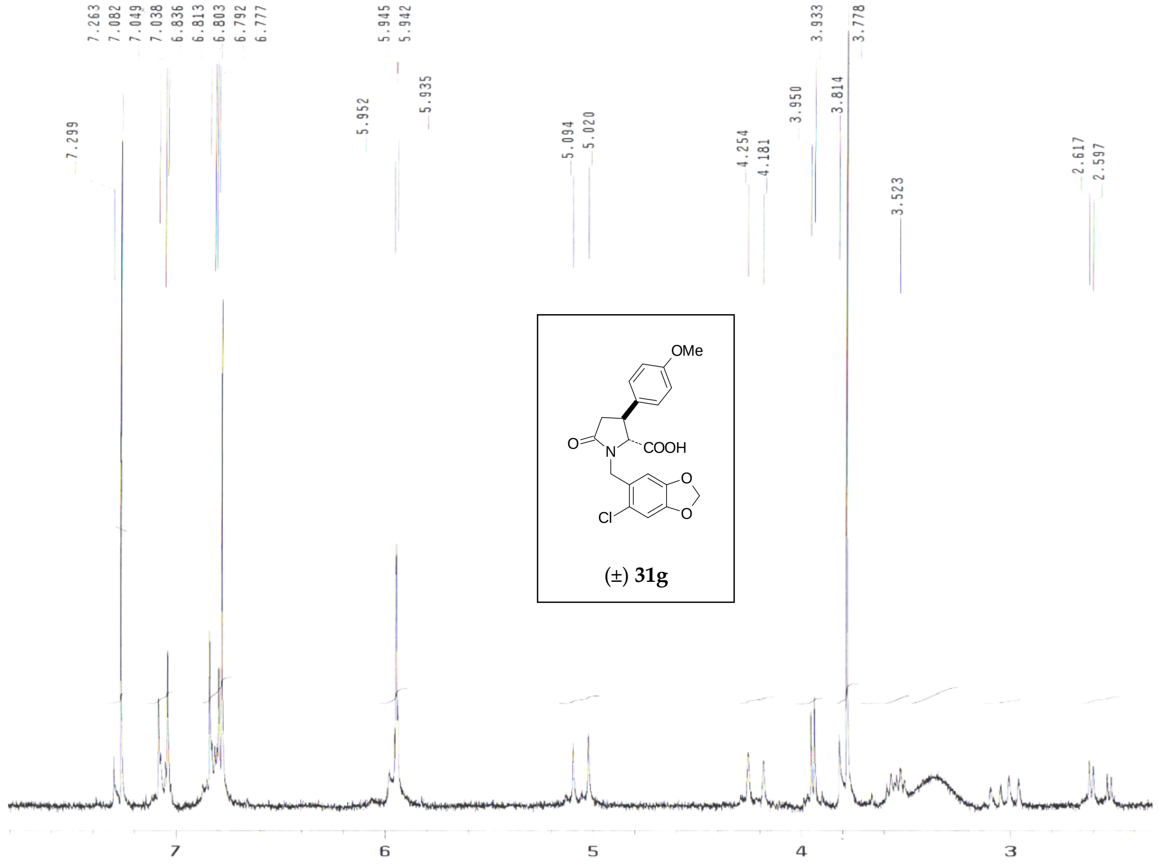

Figure S12. ^{1}H NMR spectrum of compound (±) **31g**

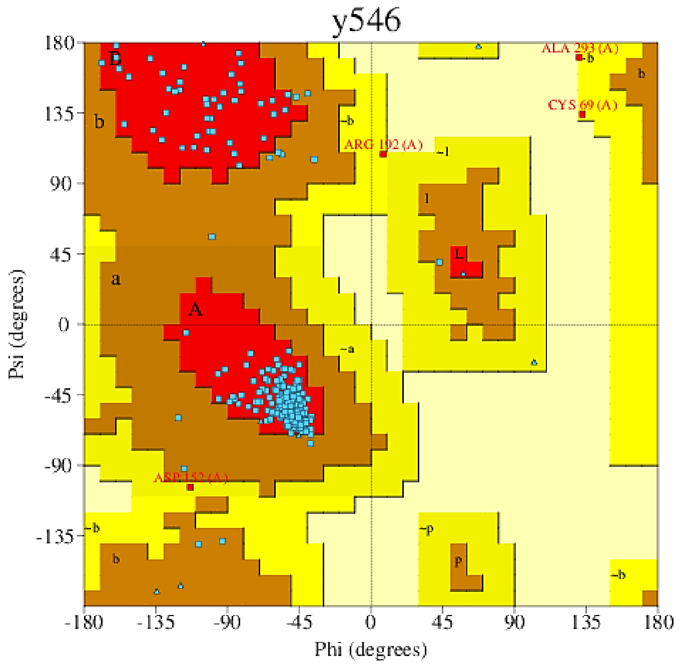

Figure S13. Ramachandran plot analysis of the final refined model. Residues in most favoured regions 279 (93.9%), residues in additional allowed regions 14 (4.7%), residues in generously allowed regions 4 (1.3%), and residues in disallowed regions 0 (0.0%).

© 2020 by the authors. Submitted for possible open access publication under the terms and conditions of the Creative Commons Attribution (CC BY) license (http://creativecommons.org/licenses/by/4.0/).
